# Functional Phosphoproteomics in Cancer Chemoresistance Using CRISPR‐Mediated Base Editors

**DOI:** 10.1002/advs.202200717

**Published:** 2022-08-31

**Authors:** Jianan Li, Jianxiang Lin, Shisheng Huang, Min Li, Wenxia Yu, Yuting Zhao, Junfan Guo, Pumin Zhang, Xingxu Huang, Yunbo Qiao

**Affiliations:** ^1^ School of Life Science and Technology ShanghaiTech University Shanghai 201210 China; ^2^ Zhejiang Lab Hangzhou Zhejiang 311121 China; ^3^ Ninth People's Hospital Shanghai Jiao Tong University School of Medicine Shanghai 200125 China; ^4^ Shanghai Institute of Precision Medicine Shanghai 200125 China; ^5^ Precise Genome Engineering Center School of Life Sciences Guangzhou University Guangzhou 510006 China; ^6^ Zhejiang Provincial Key Laboratory of Pancreatic Disease The First Affiliated Hospital and Institute of Translational Medicine Zhejiang University School of Medicine Hangzhou 310029 China

**Keywords:** 5‐fluorouracil (5‐FU), base editors, functional phosphoproteomics, RSK2, screen

## Abstract

Selective inhibition of targeted protein kinases is an effective therapeutic approach for treatment of human malignancies, which interferes phosphorylation of cellular substrates. However, a drug‐imposed selection creates pressures for tumor cells to acquire chemoresistance‐conferring mutations or activating alternative pathways, which can bypass the inhibitory effects of kinase inhibitors. Thus, identifying downstream phospho‐substrates conferring drug resistance is of great importance for developing poly‐pharmacological and targeted therapies. To identify functional phosphorylation sites involved in 5‐fluorouracil (5‐FU) resistance during its treatment of colorectal cancer cells, CRISPR‐mediated cytosine base editor (CBE) and adenine base editor (ABE) are utilized for functional screens by mutating phosphorylated amino acids with two libraries specifically targeting 7779 and 10 149 phosphorylation sites. Among the top enriched gRNAs‐induced gain‐of‐function mutants, the target genes are involved in cell cycle and post‐translational covalent modifications. Moreover, several substrates of RSK2 and PAK4 kinases are discovered as main effectors in responding to 5‐FU chemotherapy, and combinational treatment of colorectal cancer cells with 5‐FU and RSK2 inhibitor or PAK4 inhibitor can largely inhibit cell growth and enhance cell apoptosis through a RSK2/TP53BP1/*γ*‐H2AX phosphorylation signaling axis. It is proposed that this screen approach can be used for functional phosphoproteomics in chemotherapy of various human diseases.

## Graphical abstract

A high‐throughput strategy for identifying functional phosphorylation sites involved in 5‐fluorouracil (5‐FU) resistance is introduced using clustered regularly interspaced short palindromic repeats (CRISPR)‐mediated cytosine base editor (CBE) and adenine base editor (ABE), by mutating phosphorylated amino acids with guide RNA libraries, and RSK2 and PAK4 kinases are identified as main effectors in responding to 5‐FU chemotherapy for colorectal cancer.

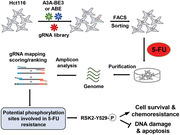

## 1 Introduction

Post‐translational modifications (PTMs) play pivotal roles in various cellular activities through structural and functional changes, which are generally reversible and pervasive.^[^ ^1^ ^]^ Among over 200 types of PTMs, phosphorylation is one of the most extensively studied PTM that orchestrates a variety of cellular functions such as cell proliferation, differentiation, apoptosis, and cellular response upon signaling stimulation and stresses.^[^ ^2^ ^]^ Till now, over 30 000 phosphorylation sites have been identified and phosphorylation process shows its involvement in almost every cellular process.^[^ ^3^ ^]^ Threonine, serine, and tyrosine are the most commonly phosphorylated amino acids, and threonine contributes to the majority of phosphorylation events.^[^ ^4^ ^]^ Protein kinases and phosphatases are the enzymes that phosphorylate and dephosphorylate their substrates respectively.^[^ ^5^ ^]^

Phosphorylation is essential for normal cellular processes and the deregulated phosphorylation events always prime the alterations of structural, functional, and regulatory proteins and attribute the subsequent deregulated signaling transduction in the manifestation of diseases, such as cancer.^[^ ^6^ ^]^ Mutations in kinases and phosphatases disrupt the cellular derangements or reprogramming of cancer‐associated proteins, which have been considered as the main cause of carcinogenesis.^[^ ^2^, ^7^ ^]^ Notably, protein kinases have become the most potential therapeutic targets for cancer therapy by using ATP analogs, monoclonal antibodies, or small molecule antagonists. For instance, Src‐FAK inhibitors, PI3K/mTOR inhibitors, MAPK inhibitors, GSK3*β*/PKD1 inhibitors, cyclin‐dependent kinase inhibitors, NF‐*κ*B inhibitors, etc., display extraordinary effects in suppressing cell growth and inducing apoptosis and have been subjected to clinical drug development for cancer therapy at various stages.^[^ ^8^ ^]^

Currently, broad‐spectrum anti‐cancer drugs that can induce DNA damage response inhibition or checkpoint suppression, such as 5‐fluorouracil (5‐FU), paclitaxel, resveratrol, cisplatin, irinotecan, and etoposide phosphate, are widely used for chemotherapy in multiple cancer treatment.^[^ ^9^ ^]^ Although the clinical applications of these drugs have improved the survival of specific kind of cancer patients, but they can cause some unpleasant side effects by targeting normal health cells.^[^ ^10^ ^]^ Thus, targeted drugs including tyrosine kinase inhibitor (TKI) imatinib, erlotinib, and nivolumab, which specifically act on cancer cells, have emerged as an attractive strategy in clinical cancer treatment combined with broad‐spectrum anti‐cancer drugs and immunotherapeutic regimens.^[^ ^9e^ ^]^ However, the frequent emergence of drug resistance for both routine chemotherapy and targeted therapy severely compromises their anticancer efficacies, the underlying mechanisms of which are diverse and complex. In addition to some genetic mutations conferring drug resistance, alterations in deregulated protein kinase activity and phosphorylation pathways upon chemotherapy and altered DNA damage responses may result in serious alternative pathways and signaling cascades during drug influx, sequestration, and response to adapt tumor microenvironment and to escape apoptosis‐inducing mechanisms.^[^ ^11^ ^]^ Identification of substitutive signaling pathways as well as distinct inhibitors for desensitizing chemoresistant cells will be of great help for poly‐pharmacological therapy. It has been proposed that altered regulation of nucleotide metabolism, amino acid metabolism, cytoskeleton organization, and oxygen metabolism may attribute to the chemoresistance in 5‐FU treatment for colon cancer cells.^[^ ^12^ ^]^ However, it remains largely unclear whether protein kinase‐mediated phosphorylation pathway is involved in 5‐FU resistance formation.

Here, we use the CRISPR‐mediated cytosine and adenine base editors (CBEs and ABEs), which have been used for saturating variant screens and functional assessment of human nucleotide variants^[^ ^13^ ^]^ and for perturbing yeast proteomes at single residue resolution,^[^ ^14^ ^]^ to screen functional phosphorylation sites in 5‐FU chemoresistance by introducing substitutions for phosphorylated amino acids in a high‐throughput manner. Through screen and subsequent validation, we reveal substrates of RSK2 and PAK4 kinases as main effectors in responding to 5‐FU chemotherapy, and combinational treatment of colon cancer cells with 5‐FU and RSK2 inhibitor or PAK4 inhibitor can largely inhibit cell growth and enhance cell apoptosis. Our study demonstrates the feasibility of BE screens for proteosome‐wide identification of essential PTM sites and catalytic enzyme‐dependent pathways in cancer chemotherapy and other disease treatment.

## 2 Results

### 2.1 CRISPR‐Dependent Base Editing Screening of Functional Phosphorylation Sites in 5‐FU Resistance

**Figure 1:**
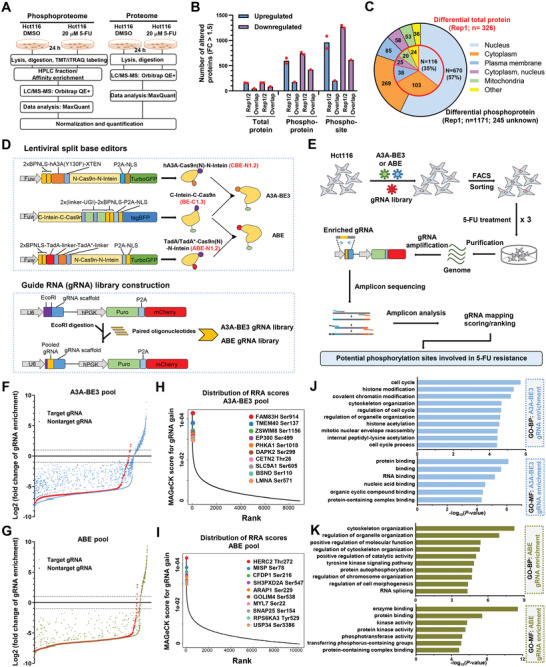
CRISPR‐mediated base editing screening of functional phosphorylation sites in 5‐FU treatment. A) The diagram describing the strategy for proteome analysis to detect total (proteome) and phosphorylation (phosphoproteome) proteins upon 5‐FU (20 × 10^−6^ m) treatment. B) Bar plot showing the number of altered proteins relative to control group with fold change (FC) > 1.5. Two replicates (Rep1 and 2) and overlapped proteins were presented for each group. C) Pie chart showing the number or proportion of differential total and phosphorylation proteins located at differential position within a cell (taking Rep1 as an example). D) The schematic diagram showing the structure of split base editor and gRNA expression vectors using a lentiviral system. E) The schematic diagram describing the procedures for BE‐mediated screening during 5‐FU (20 × 10^−6^ m) treatment and resistance formation. F,G) The distributions of gRNA enrichment (log2FC) relative to control group before 5‐FU treatment. The enrichment for gRNAs from A3A‐BE3 pool was presented in (F) and the enrichment for gRNAs from ABE‐BE3 pool was presented in (G). Red triangles indicate the enrichment of non‐targeting control gRNAs. H,I) Top depleted genes (gRNA enrichment) in 5‐FU resistant cells versus control cells before 5‐FU treatment presented as MAGeCK RPA scores using the MAGeCK algorithm. J,K) Gene ontology (GO) analysis of genes targeted by gRNAs that were significantly enriched relative to control group before 5‐FU treatment from J) A3A‐BE3 or K) ABE‐BE3 pool respectively. Two types of GO terms, including biological process (BP) and molecular function (MF), were presented.

We first examined the effect of 5‐FU on a colorectal cancer cell line, Hct116 cell, showing remarkable cell growth inhibition in a dose‐dependent manner (Figure S1A, Supporting Information). To simply evaluate the functional performance of protein phosphorylation in responding to 5‐FU treatment, we performed a proteomics profiling to quantitatively detect total proteins and phosphoproteins with or without 5‐FU treatment (**Figure** **1**A). Briefly, total protein was extracted from the two groups with equal quantity (Figure S1B, Supporting Information), and two replicates for each treatment were subjected to spectrum analysis with reproducible statistical results, such as the number of peptides, identified proteins or phosphorylation sites, and quantifiable proteins or phosphorylation sites (Figure S1C,D, Supporting Information). Moreover, protein mass and coverage distributions were generally matched with the principles of extracted proteins by using trypsin digestion and higher‐energy collisional dissociation (Figure S1E–G, Supporting Information). Correlation analysis showed that the two replicates for each treatment were highly similar from phosphoproteomics analysis and the samples with or without 5‐FU treatment were easily separated from the other group (Figure S1H, Supporting Information).

Intriguingly, only 46 upregulated and 85 downregulated total proteins were observed upon 5‐FU treatment, while there were 175 upregulated (3.8‐fold) and 420 downregulated (4.9‐fold) phosphorylated proteins identified, which contains 203 and 613 phosphorylation sites respectively (Figure 1B). Actually, there were even over 1000 differential phosphorylation sites in separate replicates (Figure 1B). Taking replicate 1 (Rep1) for proteome and phosphoproteome analysis as an example, 57% of identified phosphorylated proteins were predicted to locate in nucleus and 23% of them located in cytoplasm (Figure 1C). Similarly, the majority of total proteins also located in nucleus (35%) and cytoplasm (32%). We also calculated the sequence motif containing phosphorylation sites, revealing that prolines were frequently neighboring with phosphorylated serine and threonine (Figure S1I, Supporting Information), consistent with previous notions.^[^ ^15^ ^]^ Gene ontology (GO) analysis demonstrated that differentially expressed proteins were mainly related to nucleocytoplasmic transport, RNA splicing, and regulation of cell cycle (Figure S2A, Supporting Information), which were mainly involved in the ribosome, cytokine–cytokine receptor interaction, and p53 signaling pathways (Figure S2B, Supporting Information). Meanwhile, differentially expressed phosphoproteins were mainly associated with peptide biosynthesis, regulation of DNA metabolism, amide biosynthetic process, and negative regulation of protein binding (Figure S2C, Supporting Information), which were mainly involved in nonhomologous end joining repair, DNA replication, mRNA surveillance, and spliceosome pathways (Figure S2D, Supporting Information). The location and adjacent amino acid motif of phosphorylated proteins validate the reliability of our phosphoproteomic profiling, and these data demonstrate that phosphorylation pathways may be involved in 5‐FU chemotherapy response.

To identify specific phosphorylation sites involved in cell viability and resistance in 5‐FU chemotherapy, we meant to mutate phosphorylated amino acids (Serine, S; Threonine, T; Tyrosine, Y) using base editing tools with specific guide RNAs (gRNAs), which can target a desired loci within a defined window^[^ ^16^ ^]^ and can disrupt site‐specific post‐translational modifications.^[^ ^17^ ^]^ To assess the feasibility of BE screens for functional phosphoproteomics, we constructed Hct116 cells stably expressing BEs using lentiviral vectors. To improve the targeting efficiency restricted by the big size of lentiviral BE expression vector, the effector proteins of the base editors were split into two smaller parts (split‐A3A‐BE3^[^ ^18^ ^]^ and split‐ABE) at the position of amino acid 573/574 splitting sites for Cas9 nickase (Cas9n) (Figure 1D), and Rma intein was used for reconstituting the two BEs.^[^ ^19^ ^]^ To test the effects of position and type of nuclear localization signal (NLS) peptides, we constructed four versions of N‐intein containing CBEs and three versions of C‐intein containing CBEs with different combinations of NLS (Figure S3A, Supporting Information), which can largely improve the targeting efficiency of BEs,^[^ ^20^ ^]^ and fluorescent protein indicators for cell enrichment. Finally, we designated CBE‐N1.2 (2*BPNLS‐hA3A‐Y130F‐N‐Cas9n (1‐573)‐N‐intein‐P2A‐SV40 NLS‐TurboGFP) and BE‐C1.3 (C‐intein‐C‐Cas9n (574‐1382)‐2*UGI‐2*BPNLS‐tagBFP) as the best combination of split‐A3A‐BE3, which induced most efficient C‐to‐T conversions (Figure S3B, Supporting Information). In a similar way, we discovered ABE‐N1.2 (2*BPNLS‐TadA‐TadA*‐N‐Cas9n (1‐573)‐N‐intein‐P2A‐SV40 NLS‐TurboGFP) with an extra bipartite BPNLS relative to ABE‐N1.1 as the best split‐N‐ABE (Figure S3C, Supporting Information), which cooperated with BE‐C1.3 to reconstitute ABE and induced highest frequency of A‐to‐G conversions (Figure S3D, Supporting Information).

To enlarge the range of applications for functional phosphoproteomics using base editing screens, we designed two lentiviral gRNA libraries targeting 36883 human phosphorylation sites gathered by the PhosphoSitePlus(R) database (https://www.phosphosite.org)^[^ ^3^, ^21^ ^]^ with NGG protospacer adjacent motif (PAM) sequences. In total, 41.4% of phosphorylated amino acids (Ser, Thr, and Tyr) can be converted into other kinds of amino acids by ABE or CBE in a defined window (position 4‐8) (Figure S4A, Supporting Information). Phosphorylated amino acids are usually converted into Glu (E) or Asp (D) to mimic phosphorylation status and into Ale (A) or Phe (F) to mimic dephosphorylation status in classical biochemical assays (Figure S4B, Supporting Information).^[^ ^22^ ^]^ Theoretically, Ser, Thr, and Tyr can be converted into the other 10 types of amino acids using CBE and ABE, which may disrupt or mimic the phosphorylation process (Figure S4C, Supporting Information). Among convertible amino acids, the gRNAs inducing the alterations of acidic or basic properties for targeted amino acids were excluded, and we constructed an ABE gRNA library (10149 gRNAs targeting 10095 phosphorylation sites) and a CBE gRNA library (8885 gRNAs targeting 7779 phosphorylation sites) into a lentiviral vector for expressing gRNAs (Figures S4D and S5A, Supporting Information; Figure 1D), which contained 705 and 400 nontargeting gRNA controls (Table S1, Supporting Information).

Next, lentiviral gRNA libraries were delivered into split‐BE stably expressing Hct116 cells for a negative selection assay for cell viability and a positive selection assay for 5‐FU resistance with three rounds. Survival cells were collected for genome extraction as well as amplification and identification of enriched gRNAs by targeted deep sequencing (Figure 1E). Library‐infected cells before 5‐FU treatment were subjected to deep sequencing as negative controls, showing nearly 100% coverage and relatively uniform distributions and high homogeneity (Figure S5B,C, Supporting Information). Log‐fold changes of enriched gRNAs upon 5‐FU selection were calculated relative to nontreated controls. In relative to non‐targeting control gRNAs, 268 gRNAs from split‐A3A‐BE3 library and 160 gRNAs from split‐ABE library were enriched for more than twofold (Figure 1F,G; Table S2, Supporting Information).

Then, candidate functional phosphorylation sites were identified and ranked using the model‐based analysis of genome‐wide CRISPR/Cas9 knockout (MAGeCK) program.^[^ ^23^ ^]^ Top enriched gRNAs were predicted for targeting phosphorylation sites within FAM83H, TMEM40, ZSWIM8, EP300, PHKA1, DAPK2, CETN2, SLC9A1, BSND, LMNA, HERC2, MISP, CFDP1, SH3PXD2A, ARAP1, GOLIM4, MYL7, SNAP25, RPS6KA3, and USP34 (top 10 from split‐A3A‐BE3 library and top 10 from split‐ABE library) (Figure 1H,I). The hits predicted from significantly enriched gRNAs from split‐A3A‐BE3 library were mainly associated with cell cycle, histone modification, covalent chromatin modification, cytoskeleton organization, internal peptide‐lysine acetylation, and other cell cycle related biological processes. These proteins were mainly involved in protein binding, RNA binding, protein‐containing complex binding, and other binding‐related molecular functions (Figure 1J). The hits from significantly enriched gRNAs from split‐ABE library were mainly related to cytoskeleton organization, organelle organization, regulation of molecular function and catalytic activity, tyrosine kinase signaling pathway, and protein autophosphorylation; these proteins were also involved in molecular functions of enzyme binding, protein binding, kinase activity, and transferring phosphorus‐containing groups (Figure 1K). On the other hand, the targeting hits from significantly lost gRNAs, such as MTUS1, PLEKHA6, FIGNL1, DCDC2, SCAF1, were also mainly associated with cell cycle, chromosome organization, cytoskeleton organization, cell division, protein phosphorylation, kinase activity regulation, etc., (Figure S6A,B, Supporting Information), which may play essential roles during the loss of cell viability properties upon 5‐FU treatment. Taking these data together, it demonstrates that these significantly enriched hits might be involved in 5‐FU resistance‐linked kinase activity, protein modification, phosphorylation, and cell cycle regulation.

### 2.2 Confirmation of Top Selected Hits via Individual Amino Acid Substitutions

**Figure 2:**
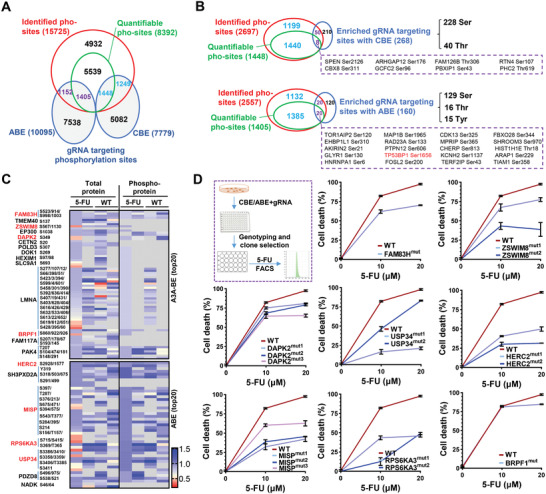
Validation of top screen hits via individual amino acid mutations. A) Veen diagram showing the number of overlapping phosphorylation sites targeted by A3A‐BE3 or ABE‐BE3 pool with that detected by mass spectrum analysis (identified or quantifiable). B) Veen diagram showing the number of overlapping phosphorylation sites targeted by significantly enriched gRNAs from A3A‐BE3 (*n* = 268) or ABE‐BE3 (*n* = 160) pool with that detected by mass spectrum analysis (identified or quantifiable) and targeted by total pools. Top enriched gRNA targeting phosphorylation sites overlapped with quantifiable phosphorylation sites are presented. C) Normalized quantification of protein peptides containing phosphorylation sites (all phosphorylation sites reported within top 20 hits from A3A‐BE3 or ABE‐BE3 pool) in total protein or phosphorylation protein analysis. Two replicates were presented for each treatment. Gray boxes indicate peptides not detected in spectrum analysis. Genes presented in top 10 (Figure 1H,I) are highlighted in blue. D) Hct116 cells were transfected with CBE or ABE tools and targeting gRNAs to establish single cell clones containing expected mutations. One, two, or three clones containing expected mutations to disrupt phosphorylation sites were subjected to apoptotic assays in responding to 5‐FU (10 × 10^−6^ or 20 × 10^−6^ m) treatment for 72 h.

Because targetable phosphorylation sites are restricted by NGG PAM sequences, we crossed the phosphorylation sites targeted by the two libraries with all identified and quantifiable phosphorylation sites pooled from all mass spectrum analyses. In total, 5254 identified phosphorylation sites and 2853 quantifiable phosphorylation sites were recovered from our screen libraries (**Figure** **2**A). Considering the relative low sensitivity of phosphoproteome,^[^ ^24^ ^]^ the real number of detected and targeted phosphorylation sties should be much larger. Among 268 enriched gRNAs from split‐CBE library (228 Ser and 40 Thr were targeted), 58 identified phosphorylation sites were recovered, including 8 quantifiable sites within SPEN, ARHGAP12, FAM126B, RTN4, CBX8, GCFC2, RBXIP1, and PHC2; among 160 enriched gRNAs from split‐ABE library (129 Ser, 16 Thr, and 15 Tyr were targeted), 40 identified phosphorylation sites were recovered, including 20 quantifiable sites within CDH13, RAD23A, PTPN12, HIST1H1E, TP53BP1, FOSL2, TIAM1, etc. (Figure 2B).

We further analyzed the relative levels of phosphorylated amino acid‐containing peptides in proteosome and phosphoproteosome analyses, demonstrating that the majority of top 20 targeted hits can be detected in total proteins and/or phosphorylated proteins in at least one replicate, indicating the possible role of these hits in a phosphorylation‐dependent manner (Figure 2C). To validate the potential role of top screened hits in 5‐FU chemoresistance, we constructed gRNAs targeting these top hits and gRNAs were cotransfected into Hct116 cells with CBE/ABE tools; single clones containing expected mutations to disturb phosphorylated amino acids were established for testing 5‐FU response. As expected, nearly all mutant clones, including FAM83H Ser914, ZSWIM8 Ser1156, DAPK2 Ser299, HERC2 Thr272, MISP Ser78, RPS6KA3 Tyr529, USP34 Ser3386, showed resistance to 5‐FU‐induced cell apoptosis, while BRPF1 mutant, which was not included in the top screen hits, showed nearly no inhibitory effect in apoptotic induction (Figure 2D; Table S3, Supporting Information). These data demonstrate the reliability of functional phosphorylation sites identified from BE screens in 5‐FU chemoresistance.

### 2.3 Top Selected Hits Mediate 5‐FU‐Induced Transcriptomic Alterations

To assess the mechanism underlying 5‐FU‐induced apoptosis, we performed RNA‐seq analysis of Hct116 cells with or without 5‐FU treatment. 5‐FU‐downregulated genes were mainly related to cell cycle progression, small molecule metabolic process, spindle organization, which were involved in mitotic prometaphase, sister chromatid cohesion, cell cycle, spindle formation, cellular amino acid metabolism, and Rho GTPases pathways (Figure S7A,B, Supporting Information). On the contrary, 5‐FU‐upregulated genes were mainly associated with response to stress, programmed cell death, apoptotic process, negative regulation of cell communication and signaling, etc.; these upregulated genes were mainly involved in Interleukin‐10 signaling, regulation of necroptotic cell death, regulated necrosis, transcriptional regulation by TP53, extrinsic apoptotic signaling pathway, regulation of peptidase activity, and other pathways (Figure S7A,B, Supporting Information).

Next, we compared the expression of wild‐type Hct116 cells with top hit mutant clones, which displayed remarkable apoptosis‐resistant phenotypes, including RPS6KA3 (encoding RSK2), FAM83H, ZSWIM8, and MISP mutants. Genes associated with response to peptide, epithelial cell proliferation, response to nutrient levels and stress, were downregulated in RSK2 mutant cells, and genes related to protein refolding, negative regulation of protein ubiquitination, regulation of localization, and protein conjugation or removal, were upregulated in RSK2 mutant cells (Figure S8A, Supporting Information).

Surprisingly, FAM83H, ZSWIM8, and MISP mutants showed 226 common downregulated genes, which were related to ER‐nucleus signaling pathway, response to epidermal growth factor, and cytokine metabolic process, as well as 63 common upregulated genes that were related to multi‐organism localization, response to dsRNA, and response to topologically incorrect protein (Figure S8B, Supporting Information). We then asked whether the dysregulated phosphorylation of identified top hits mediated the 5‐FU‐induced transcriptomic alterations, FAM83H, ZSWIM8, and MISP mutants were stimulated with 5‐FU for 24 h, and cells were subjected to bulk RNA‐seq analysis. The expression level of 71 common genes (e.g., *LCN2*, *FZD3*, *EHF*, *KRT7*, *SNAI3*, *TRIM29*, *EGLN3*) in 5‐FU‐treated mutant cells was significantly lower than that in 5‐FU‐treated wildtype Hct116 cells, and the expression of 46 common genes (e.g., *FOXD1*, *HERC5*, *CCN1*, *DLG2*, *SLC2A3*, *IFNL2*) in 5‐FU‐treated mutant cells was much higher than that in 5‐FU‐treated wildtype cells (Figure S9A,B Supporting Information). These data indicate that the disrupted phosphorylation of FAM83H, ZSWIM8, and MISP partially may mediate 5‐FU chemoresistance downstream of a same or close signaling pathway.

**Figure 3:**
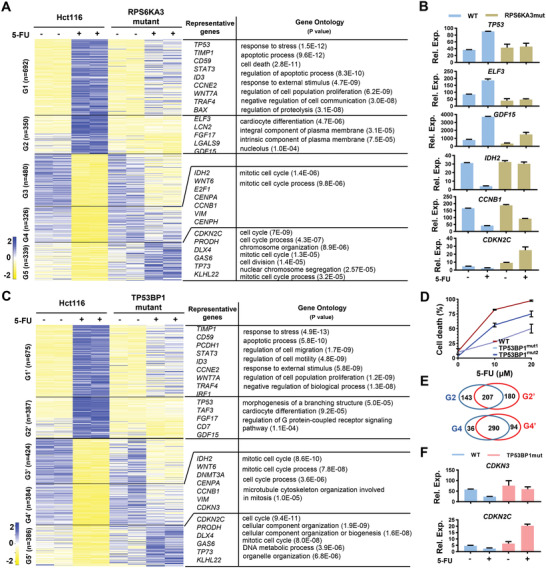
RSK2 and TP53BP1 mediate 5‐FU‐induced transcriptomic alterations. A) RNA‐seq analysis of differentially expressed genes in control (Hct116) or RPS6KA3 mutant cells without or without 5‐FU (20 × 10^−6^ m) treatment. Five group of genes (G1‐G5) with different features were presented. Representative genes and GO analysis for G1, G2, G4, and G5 groups were presented (P values for GO terms were also presented). B) Relative expression (Rel. Exp.) of *TP53*, *ELF3*, *GDF15*, *IDH2*, *CCNB1*, and *CDKN2C* was presented as FPKM in control (WT/Hct116) or RPS6KA3 mutant cells without or without 5‐FU treatment from RNA‐seq analysis. C) RNA‐seq analysis of differentially expressed genes (G1’‐G5’) in control (Hct116) or TP53BP1 mutant cells without or without 5‐FU treatment. Representative genes and GO analysis for G1’, G2’, G4’, and G5’ groups were presented (P values for GO terms were also presented). D) WT/Hct116) or TP53BP1 mutant cells were subjected to apoptotic analysis in responding to 5‐FU (10 × 10^−6^ or 20 × 10^−6^ m) treatment for 72 h. E) Veen diagram showing the number of overlapping genes between G2 versus G2’ or G4 versus G4’. F) Relative expression (Rel. Exp.) of *CDKN3* and *CDKN2C* was presented as FPKM in control (WT/Hct116) or TP53BP1 mutant cells without or without 5‐FU treatment from RNA‐seq analysis.

Subsequently, we also analyzed the 5‐FU‐induced transcriptome‐wide alterations in RSK2 mutant cells. Notably, the 5‐FU‐induced gene upregulation was significantly impaired (G1; *n* = 692) or even completely blocked (G2; *n* = 350) in RSK2 mutant cells. These genes were associated with response to stress, apoptotic process, and cell death, such as *TP53*, *WNT7A*, *STAT3*, *CCNE2*, *BAX*, *ELF3*, *LCN2*, *GDF15*, etc. (**Figure** **3**A,B). Meanwhile, the 5‐FU‐induced gene downregulation was also significantly weakened (G4; *n* = 326) or even upregulated (G5; *n* = 339) in RSK2 mutant cells. The two groups of genes are mainly related to cell cycle process, cell division, and chromosome organization, such as *IDH2*, *E2F1*, *CCNB1*, *VIM*, *CENPA*, *CDKN2C*, *TP73*, *KLHL22*, etc. (Figure 3A,B). These data together demonstrate that RSK2 mutation antagonizes 5‐FU‐induced cell apoptosis and cell cycle arrest, further indicating that RSK2 phosphorylation mediates the transcriptomic program during 5‐FU chemotherapy.

Among the downstream effector of 5‐FU treatment, TP53 was significantly upregulated by 5‐FU, which was blocked by RSK2 mutation (Figure 3B). Coincidently, TP53BP1 Ser1656 was also identified as one of the top hits in split‐ABE library (Figure 2B), which cooperated with TP53 in controlling anti‐tumorigenic activities during DNA repair, DNA damage response, and tumor suppression.^[^ ^25^ ^]^ TP53BP1 has been reported to be phosphorylated in response to double‐stranded DNA breaks and is recruited to DNA lesion sites with *γ*‐H2AX phosphorylation to promote the nonhomologous end‐joining (NHEJ)‐mediated DNA repair.^[^ ^26^ ^]^ We hypothesized that RSK2 phosphorylation may act upstream of TP53/TP53BP1 phosphorylation signaling pathway to mediate 5‐FU‐induced DNA damage and apoptotic response. As expected, TP53BP1 Ser1656 mutant cells exhibited highly similar transcriptomic alterations upon 5‐FU stimulation (Figure 3C), accompanied by anti‐apoptosis phenotype (Figure 3D). Moreover, the upregulation of 387 genes (G2’) induced by 5‐FU was abolished by TP53BP1 mutation, which also blocked the downregulation of 384 genes (G4’) induced by 5‐FU (Figure 3C). Intriguingly, the majority of differentially expressed genes for 5‐FU‐treated TP53BP1 mutant cells were overlapped (*n* = 207 for G2 versus G2’; *n* = 290 for G4 versus G4’) with that from 5‐FU‐treated RSK2 mutant cells (Figure 3E). For instance, the 5‐FU‐induced upregulation of *TP53* and *GDF15* was diminished in both TP53BP1 and RSK2 mutant cells; the 5‐FU‐induced downregulation of *CDKN3* and *CDNK2C*, which act as Cyclin‐dependent kinase inhibitors to promote cell cycle progression and tumorigenesis, was diminished in both TP53BP1 and RSK2 mutant cells, with even much higher expression upon 5‐FU stimulation (Figure 3B,C,F). Thus, we propose that RSK2/TP53BP1 signaling axis functions downstream of 5‐FU to induce cell cycle arrest and apoptosis in a phosphorylation‐dependent cascade.

### 2.4 Synergetic Lethal Effect of 5‐FU and RSK2 or PAK4 Inhibitors on Colorectal Cancer Cells

The overall response rate for 5‐FU is only 11% in the treatment of patients with metastatic colorectal cancer,^[^ ^27^ ^]^ and oxaliplatin, leucovorin, irinotecan, and other broad‐spectrum anti‐cancer agents have been applied for the potentiation of 5‐FU chemosensitivity.^[^ ^28^ ^]^ However, chemoresistance to anti‐cancer drugs is a major barrier in cancer treatment with innate and acquired resistant manifestation. Acquired resistance to one drug often confers resistance to other anti‐cancer drugs even with different mechanisms of action, suggesting that cancer cells share a common mechanism for resistance formation.^[^ ^29^ ^]^

**Figure 4:**
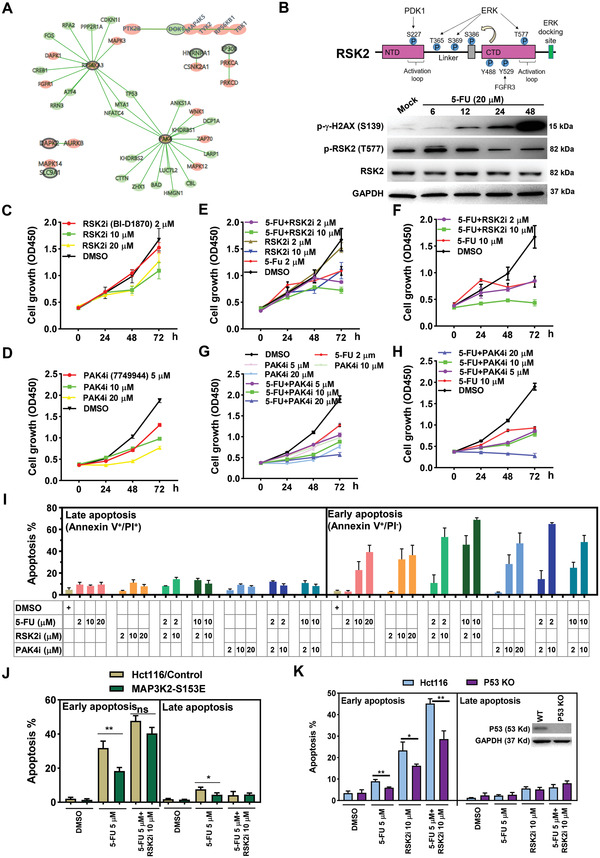
RSK2 or PAK4 inhibitors enhancer 5‐FU‐induced cell growth inhibition and apoptosis in colorectal cancer cells. A) Top 20 screen hits were subjected to phosphorylation network analysis using an online tool (http://phosphorylationnetworks.org). Black circled genes were from screen hits, genes with pink background represent known kinases, and genes with green background represent classical downstream targets. B) The upper panel displays the phosphorylation sites and functional domains (NTD, NH2‐terminal kinase domain; CTD, C‐terminal domain) identified within RSK2 protein as well as their catalyzing kinases (PDK1, ERK, FGFR3). The upper panel showing the results from western blot analysis of total RSK2, phosphorylated RSK2 at Thr577 (p‐RSK2 T577), and GAPDH expression in mock or 5‐FU‐treated Hct116 cells (20 × 10^−6^ m for 6, 12, 24, and 48 h). C,D) Relative cell growth rates of Hct116 cells treated with increasing concentrations of RSK2 inhibitor (RSK2i) or PAK4 inhibitor (PAK4i). E–H) Relative cell growth rates of Hct116 cells co‐treated with 5‐FU (2 × 10^−6^ or 10 × 10^−6^ m) and RSK2i or PAK4i. I) Apoptotic analysis of Hct116 cells co‐treated with 5‐FU (2 × 10^−6^ or 10 × 10^−6^ m) and RSK2i or PAK4i. Proportions of early (Annexin V^+^/PI^–^) or late (Annexin V^+^/PI^+^) apoptotic cells were presented. J) Apoptotic analysis of Hct116 cells expressing control vector or MAP3K2‐S153E. Cells were treated with 5‐FU (5 × 10^−6^ m) or 5‐FU and RSK2 inhibitor (10 × 10^−6^ m) combinations for 72 h, and the proportions of early and late apoptotic cells were determined. K) Wildtype or P53 knockout (P53 KO) Hct116 cells were treated with 5‐FU (5 × 10^−6^ m), RSK2 inhibitor (10 × 10^−6^ m), or their combinations. Apoptotic assays were performed at 48 h. Western blot analysis was conducted to detect the expression of P53 in wildtype and P53 KO cells.

In our phosphorylation targeting screening, we observed a RSK2/TP53BP1 signaling cascade as well as an unknown mechanism of FAM83H, ZSWIMM8, and MISP involved in acquiring 5‐FU chemoresistance (Figure 3; Figure S8, Supporting Information). Considering the kinases‐dependent phosphorylation process,^[^ ^5^ ^]^ we asked whether the top screen hits were involved in some common kinases‐regulated signaling pathways. Therefore, top screen hits were subjected to phosphorylation network analysis,^[^ ^30^ ^]^ and RSK2, PAK4, DAPK2, HNRNPA1, and EP300 signaling pathways were identified as sub‐signaling pathway centers (**Figure** **4**A). Interestingly, RSK2 and PAK4 displayed as central regulators upstream of multiple phosphorylation substrates, and they regulated common targets, including TP53, MTA1, and NFATC4 (Figure 4A). Recently, it has been reported that feedback activation of the EGFR/PAK2/ERK5 signaling limits the sensitivity of liver cancer cells to lenvatinib chemotherapy.^[^ ^31^ ^]^ Coincidently, FGFR3 activates RSK2 through tyrosine 529 phosphorylation of RSK2 to activate MEK/ERK pathway during hematopoietic transformation (Figure 4B).^[^ ^32^ ^]^ We propose that RSK2 and PAK4 may integrate at the TP53^[^ ^33^ ^]^ and ERK pathway^[^ ^34^ ^]^ to mediate 5‐FU chemoresistance. Intriguingly, both RSK2 and PAK4 were highly expressed in multiple cancer types (Figure S10A–D, Supporting Information). Moreover, we observed a transient upregulation and subsequent downregulation of RSK2 phosphorylation at Thr577 by 5‐FU, which was accompanied by the accumulation of *γ*‐H2AX phosphorylation in a time‐dependent manner^[^ ^35^ ^]^ (Figure 4B). It showed that the downregulation of RSK2 phosphorylation was positively correlated with the upregulation of apoptosis‐linked *γ*‐H2AX phosphorylation. We propose that the activation or retainment of RSK2 phosphorylation may confer to cell survival upon 5‐FU treatment, possibly regarding the RSK2/TP53BP1/*γ*‐H2AX signaling axis.^[^ ^36^ ^]^

Next, we asked whether blockade of RSK2 or PAK4 activation can sensitize colorectal cancer cells to 5‐FU treatment. Similar to 5‐FU effect on cell growth (Figure S1A, Supporting Information), RSK2 and PAK4 inhibitors (IC50 = 12.43 µM mL^−1^ for LCH‐7749944 and IC50 = 16.28 µM mL^−1^ for BI‐D1870; Figure S11A,B, Supporting Information) suppressed cell proliferation in a dose‐dependent manner, especially at 48 and 72 h (Figure 4C,D). When both inhibitors were combined with 5‐FU to treat Hct116 cells, we found that the inhibitory effects of 5‐FU on cell growth at 2 × 10^−6^ or 10 × 10^−6^ m were significantly enhanced by RSK2 or PAK4 inhibitors in short‐term and long‐term assays (Figure 4E–H). We also tested the synergy between 5‐FU and RSK2 or PAK4 inhibitors on cell apoptosis detected by Annexin V/PI assays (Figure S11C, Supporting Information). Overall, both inhibitors enhanced 5‐FU‐induced early (Annexin V+/PI‐) and late (Annexin V+/PI+) apoptosis (Figure 4I; Figure S11D, Supporting Information), accompanied by the upregulation of *γ*‐H2AX phosphorylation and the downregulation of RSK2 phosphorylation upon RSK2 inhibitor combination (Figure S11E, Supporting Information). Relatively, the proportion of 5‐FU‐induced early apoptotic cells was increased more than late apoptotic cells upon RSK2 or PAK4 inhibitor combinations (Figure 4I).

We also verified the potential role of RSK2 and PAK4 in 5‐FU chemoresistance in DLD‐1 colon cancer cells. As expected, cell growth inhibition was observed upon 5‐FU, RSK inhibitor, or PAK4 inhibitor treatment in a dose‐dependent manner (Figure S12A–C, Supporting Information). We noticed that the inhibitory effect was observed at 96 h in DLD cells, which was delayed comparing to Hct116 cells. Moreover, combinational treatment with RSK2 inhibitor or PAK4 inhibitor can significantly sensitize DLD1 cells to 5‐FU stimulation (Figure S12D,E, Supporting Information), further validating the reliability of our BE screen strategy. To extend our observation to clinical applications, two clinical inhibitors, BIX02565 (a RSK2 inhibitor) and Kpt9274 (an orally bioavailable PAK4 inhibitor), were utilized for combinational treatment with 5‐FU in Hct116 cells. Consistent with the results from BI‐D1870 and LCH‐7749944 (Figure 4I), BIX02565 and Kpt9274 displayed a synergetic effect on 5‐FU‐induced apoptosis, although the responsive concentrations of these two inhibitors were much higher than BI‐D1870 and LCH‐7749944 (Figure S12F, Supporting Information).

To further investigate PAK4‐linked ERK and RSK2‐linked P53 signaling pathways in 5‐FU chemoresistance, we disturbed ERK and P53 signaling pathways by genetic manipulation or chemical inhibitors. Similar to the responsiveness for PAK4 inhibitors (Figure 4I), ERK inhibitor Ulixertinib strongly induced cell apoptosis and enhanced 5‐FU‐elicited effects in both Hct116 and DLD1 cells (Figure S13A,B, Supporting Information). On the contrary, overexpression of a constitutively active form of MAP3K2 (MAP3K2‐S153E), which acts upstream of ERK5 pathway, antagonized 5‐FU‐induced cell apoptosis, while this antagonizing effect was largely diminished upon RSK2 inhibition (Figure 4J), suggesting a functional correlation between ERK and RSK2 signaling pathways. We also tested the 5‐FU responsiveness in wildtype (WT) and P53 knockout (P53 KO) Hct116 cells. We found that 5‐FU‐, RSK2 inhibitor‐, or their combination‐induced apoptosis was significantly decreased in P53 KO cells (Figure 4K), demonstrating that the upregulation of TP53, which was diminished in RSK2 mutant cells (Figure 3B), may act as a pro‐apoptotic upon 5‐FU treatment. Collectively, these data suggest that RSK2 or PAK4 phosphorylation mediates resistance to 5‐FU chemotherapy and that suppression of RSK2 or PAK4 activation in combination with 5‐FU displays a synergistic inhibitory effect on cell growth and apoptosis in colorectal cancer cells, involving ERK and P53 signaling pathways (Figure S13C, Supporting Information).

## 3 Discussion

Anticancer drugs, such as 5‐FU, cisplatin, fludarabine, paclitaxel, and camptothecin, are widely used in chemotherapeutics by targeting DNA synthesis and cell cycle progression, while chemoresistance emerges as a major barrier in cancer treatment with innate and acquired resistant manifestation to attribute to low response rates.^[^ ^8^ ^]^ Thus, the contemporary cyclin‐dependent kinase inhibitors, such as EGFR, PARP, VEGF, CDK4/6 inhibitors, etc., have been combinationally used for cancer therapy in clinical use or trials to antagonize chemoresistance,^[^ ^37^ ^]^ highlighting the potential role of kinase‐dependent phosphorylation pathways in chemoresistance formation (Figure 1A–C). In the present study, we utilized CRISPR‐mediated base editors for screening functional phosphorylation sites involved in 5‐FU chemoresistance and discovered several substrates of RSK2 and PAK4 kinases as main effectors in responding to 5‐FU chemotherapy, inhibition of which enhanced 5‐FU‐induced cell growth inhibition and apoptosis.

For base editing‐based screen, we need to consider the targeting scope, specificity, and efficiency for phosphorylation site‐targeting library design to extremely increase the reliability of screened hits. Because the targeted amino acid is fixed for a specific protein, the on‐targeting efficiency and off‐targeting potential are less considered. To elevate the targeting efficiency as far as possible, we apply a split strategy for BEs delivery, which is much more efficient than full‐length BEs (Figure 1; Figure S3, Supporting Information). Moreover, APOEC3A‐fused BE3 is utilized for CBE screen, because APOEC3A displays higher editing efficiencies and lower bias,^[^ ^18^, ^38^ ^]^ although its wider targeting window^[^ ^18^ ^]^ may generate bystander mutations out of the expected targeting window. However, all tested top hits exhibit an anti‐apoptotic phenotype, validating the reliability of our functional phosphoproteomics strategy (Figure 2).

There are three points that can be optimized for our targeting library design. First, only targetable phosphorylation sites with NGG PAMs were incorporated (Figure S4, Supporting Information). We can construct a larger library to target all targetable phosphorylation sites without PAM restriction using PAMless SpRYCas9‐based BEs.^[^ ^39^ ^]^ Second, the mutated products by CBE and ABE can only disrupt the phosphorylation status (Figure S4C, Supporting Information), whereas they cannot generate “E” or “D” to mimic stably phosphorylated status for phosphorylation‐dependent gain‐of‐function analysis. In addition, some amino acid conversions may function through conformational change but independent of phosphorylation process. Prime editing tools‐based screen can be anticipated to cover all phosphorylation sites with gain‐of‐phosphorylation and loss‐of‐phosphorylation analysis.^[^ ^40^ ^]^ In a recent report, targeted histone phosphorylation has been achieved using a CRISPR/Cas9‐based chromatin kinase,^[^ ^41^ ^]^ and the targeted phosphorylation on non‐histone proteins will be anticipated. Third, the overlapping ratio between targeted phosphorylation sites and identifiable or quantifiable sites is not high enough (Figure 2A). For targeted screen, proteomics and phosphoproteomics can be performed and then sublibraries can be constructed to target identifiable or differential phosphorylation sites, which may help to identify functional phosphorylation sites in a specific biological process.

Consistent with the notion that cancer cells may share a common mechanism for resistance to different anti‐cancer drugs,^[^ ^29^ ^]^ among top screen hits, RSK2 and TP53BP1 mutants restrain 5‐FU‐induced apoptosis by antagonizing 5‐FU‐triggered transcriptomic alterations, with a high proportion of overlapping target genes (Figure 3). Thus, we propose a RSK2/TP53BP1/*γ*‐H2AX phosphorylation signaling cascade during 5‐FU‐induced DNA damage response and chemoresistance formation (Figures 3 and 4). The cells with high RSK2 phosphorylation upon 5‐FU treatment, which is negatively correlated with *γ*‐H2AX phosphorylation, may acquire resistance to growth inhibition and apoptosis. Thus, mutation of RSK2 phosphorylation site can antagonize 5‐FU‐induced apoptotic effect and transcriptomic alterations in colorectal cancer cells (Figures 2 and 3). In addition, inhibition of RSK2 or PAK4 enhances 5‐FU‐triggered colorectal cancer cell apoptosis (Figure 4), which is consistent with the finding that RSK2 knockdown induces apoptosis in human myeloma cells.^[^ ^32^ ^]^ Considering the role of EGFR/PAK2/ERK5 signaling axis in resistance to lenvatinib and the role of RSK2 in EGF‐activated histone H3 phosphorylation and inactive ERK binding to RSK2,^[^ ^31^, ^42^ ^]^ we postulate that RSK2/TP53BP1/*γ*‐H2AX and PAK4/ERK may confer to 5‐FU chemoresistance through a same signaling pathway or an integrated interaction network. Coordinately, ERK inhibition enhances 5‐FU‐induced apoptosis while P53 knockout confers resistance to 5‐FU‐ and RSK inhibitor‐induced cell death^[^ ^43^ ^]^ (Figure 4), highlighting the essential role of ERK and P53 signaling pathways in 5‐FU chemoresistance. However, the detailed coordinative relationship between PAK4/ERK, RSK2/TP53BP1, and P53 pathways needs further investigation in the future. Combing these findings together, it underlies the essential role of RSK2 and PAK4 in carcinogenesis and chemoresistance, and both factors may become a cancer prevention target for poly‐pharmacological or combinational therapies.^[^ ^44^ ^]^ Our successful identification of RSK2 and PAK4 as combinational therapeutic targets also proves the feasibility of BE‐mediated screens for characterizing functional phosphorylation sites that can be further applied for discovering key upstream kinases as perturbing targets in a specific process.

In sum, we establish a BE‐based screen system for identifying functional phosphorylation sites involved in 5‐FU chemotherapy response and reveal RSK2/TP53BP1/*γ*‐H2AX and PAK4/ERK signaling pathways as anti‐chemoresistance targets, blockade of which enhances cell growth inhibition and apoptosis (Figure S13C, Supporting Information). This system enables phenotypic characterization of disease‐associated phosphorylation sites, some of which are clinically mutated and relevant to therapy efficacy and disease progression. The present study puts the detection of protein modification sites forward to high‐throughput functional analysis, and this approach can be extended to functional characterization of other post‐translational modifications. Future improvements in targeting scope and precision will increase the probability for discerning whole proteosome‐wide modification sites, facilitating targeted and poly‐pharmacological therapies in precision medicine development.

## 4 Experimental Section

### Cell Culture and Viral Production

Human colorectal cancer Hct116 and DLD1 cells were cultured in RPMI‐1640 medium supplemented with 10% FBS and 100 units per ml penicillin and streptomycin. Human embryonic kidney (HEK) 293T (HEK293T) cells were grown in Dulbecco′s modified eagle medium in 10% FBS and 100 units per ml penicillin and streptomycin. Both cells were purchased from ATCC and cultured at 37 °C with 5% CO2. Lentivirus was produced by transfecting HEK293T cells with transfer plasmids and standard packaging plasmids using standard calcium phosphate transfection method.

### Vectors and Reagents

The synthesized DNA oligos for sgRNA‐expressing plasmid construction were annealed and cloned into pGL3‐U6‐sgRNA‐PGK‐mCherry, with EGFP replaced by mCherry in pGL3‐U6‐sgRNA‐PGK‐EGFP (Addgene #107721). Synthesized oligos for gRNA construction are shown in Table S4 (Supporting Information). pLenti‐gRNA expression vector (Hongxun Technology, Suzhou, China) was used for gRNA library construction. Customized gRNA cassette was driven by U6 promoter and mCherry expression was driven by pGK promoter as a transfection indicator. For constructing lentiviral expression vector expressing split base editors, full‐length A3A‐BE3 and ABE (ABE7.10) constructs^[^ ^45^ ^]^ were used as templates for PCR amplification of partial coding sequences of base editors. split BE products were amplified by Phanta Max Super‐Fidelity DNA Polymerase (P505, Vazyme) using paired primers and PCR primers were shown in Table S5 (Supporting Information). Amplified split A3A‐BE3/ABE products as well as full‐length A3A‐BE3/ABE were constructed into a lentiviral vector (Fuw‐TRE‐BamH1‐P2A‐nls‐TuroboGFP; linearized by BamH1 digestion) using the ClonExpress II One Step Cloning kit (C112‐02, Vazyme). N‐terminal and C‐terminal part of base editors were split at codon 573/574 of nCas9 using Rma intein, with nuclear localization signal (NLS) and TurboGFP or tagBFP as selection markers. Different versions of split base editors (BE‐N1.1, N1.2, N1.3, N1.4; ABE‐N1.1, N1.2; BE‐C1.1, C1.2, C1.3) were constructed with distinct position and type of nuclear localization signal (NLS) peptides as well as fluorescent protein indicators (Figure S3, Supporting Information). MAP3K2‐S153E plasmid was purchased from MiaoLing Plasmid Platform, and it was sub‐cloned into the lentiviral vector for establishing a stable Hct116 line expressing MAP3K2‐S153E with the help of tTA activation. Chemical reagents, including 5‐FU (HY‐90006), BI‐D1870 (HY‐10510), LCH‐7749944 (HY‐125035), BIX02565 (HY‐16104), Ulixertinib (HY‐15816), and KPT9274 (HY‐12793), were purchased from MedChemExpress (MCE, USA).

### Design of Genome‐Scale gRNA Libraries of CBE/ABE Targeting Phosphorylation Sites

36883 human phosphorylation sites gathered by the PhosphoSitePlus(R) database (https://www.phosphosite.org) were collected, and gene annotations were retrieved from the UCSC hg38 genome. All possible gRNAs targeting these phosphorylation sites with “NGG” PAMs (“N” is any nucleobase) were considered, and the targeted cytosines or adenines were located at positions 4–8 of gRNAs (the distal position from PAM is defined as position “1”). Ser, Thr, and Tyr amino acids could be converted into Pro, Gly, Ala, Cys, Phe by gRNA library for split A3A‐BE3; Ser, Thr, and Tyr amino acids could be converted into Phe, Leu, Asn, Pro, Ile, Met by gRNA library for split ABE. The final split A3A‐BE3 gRNA library contained 8885 gRNAs targeting 7779 phosphorylation sites and 400 non‐targeting gRNAs. The final ABE gRNA library contained 10149 gRNAs targeting 10095 phosphorylation sites and 705 non‐targeting gRNAs used as negative controls. Pooled gRNA oligonucleotides were synthesized and cloned into pLenti‐U6‐gRNA‐pGK‐Cherry expression vector (Hongxun technology; Suzhou, China). Next‐generation sequencing (NGS) was performed to detect library coverage and distributions.

### Lentivirus Production

Pooled lentiviral library and lentivirus for split‐BE overexpression were produced in HEK293T cells. 24 h before transfection, HEK293T cells were seeded into a 10‐cm culture dish and the transfection was performed using conventional calcium phosphate transfection methods. A DNA mixture of lentiviral transfer vector (10 µg), pCMV_Delta8.9 (6 µg), pCMV_VSVG (4 µg) was prepared. After incubation for 8 h, the media was replaced with fresh media. Virus was harvested twice at 48 h and 72 h after media change and concentrated by ultracentrifugation at 27 000 rpm for 2 h at 4 °C. Virus was dissolved in 200–500 µL DMEM medium. To determine lentiviral titer for transductions, cell lines were transduced into 12‐well plates with a range of virus volumes (e.g., 0, 0.001, 0.01, 1, 10 virus) with 1 × 10^5^ cells per well in the presence of polybrene and mixture was incubated for 4–6 h. Three days post‐transduction, the transduction efficiency was measured by detecting the proportion of fluorescent‐positive cells. A viral dose resulting in 30%–70% transduction efficiency was used for the following library screening.

### Pooled Screen upon 5‐FU Treatment

For pooled screen, Hct116 cells were infected with lentivirus containing N‐terminal (GFP) and C‐terminal (tagBFP) split A3A‐BE3 or split‐ABE. Meanwhile, cells were also infected with lenti‐tTA to activate TRE‐driven split base editors. Then infected cells were subjected to cell sorting with fluorescence‐activated cell sorted (FACS) (BD FACSAria III), and GFP/tagBFP double positive cells were enriched for cell expansion. Then split BE‐expressing Hct116 cells (1 × 10^7^) were infected with lentiviral gRNA libraries targeted by split A3A‐BE3 or split‐ABE separately at a low multiplicity of infection (MOI: 0.3–0.7), to obtain enough cells to achieve an average number of 500 transduced cells per gRNA (20‐50% transduction efficiency). During lentiviral infection, mixture of cells, lentivirus, and polybrene at 0.5 µg mL^−1^ was incubated for 6–8 h. After infection for 7–10 d, infected cells were subjected to 5‐FU (10 × 10^−6^ m; HY‐90006, MCE) or DMSO treatment for 3 d, allowing gRNAs to enrich or deplete; about 20% alive cells were passaged for expansion under normal culture conditions. After expansion for 8–10 d, cells were subjected to another two rounds of 5‐FU treatment. At the beginning of 5‐FU treatment, infected cells were collected as a control. The cells after three rounds of 5‐FU treatment were collected for genomic DNA (gDNA) extraction. Three replicates were collected for each group of experiment.

### Genomic DNA Extraction and Sequencing

Genomic DNA from post‐screening cells was extracted using DNA Isolation Kit (Tiangen, DP304) according to the manufacturer's instructions. PCR amplification for base editor screens was performed with Phanta Max Super‐Fidelity DNA Polymerase (P505, Vazyme) using barcoded primers, as listed in Table S6 (Supporting Information). PCR amplifications were performed in 200 µL reactions and 4 µg DNA was added to the reactions. A 200 µL reaction was aliquoted into 4 pieces of 50 µL reactions. PCR cycling conditions were as follows: an initial 2 min at 95 °C; followed by 20 s at 95 °C, 20 s at 60 °C, 30 s at 72 °C, for 25 cycles; and a final 7 min extension at 72 °C. PCR products were purified with PCR clean‐up kit (Axygen). PCR products with different barcodes were pooled together for targeted deep sequencing^[^ ^46^ ^]^ on Illumina Nextseq 500 (2×150 PE) platform at the Novogene Bioinformatics Institute, Beijing, China.

### MAGeCK Analysis of gRNA Enrichment

The screening data were analyzed using Count_space.py and the pipeline MAGeCK (ver. 0.5.6). In A3A‐BE3‐based and ABE‐based screens, 400 and 705 nontargeting gRNAs were used as non‐target controls. The gRNA sequencing data before 5‐FU treatment served as a negative control for MAGeCK analysis. CRISPResso2 (ver. 2.0.30) was used to process all targeted deep sequencing reads for acquiring specific sequencing reads for each gRNA.

### Establishment of Mutant Cell Lines Using BEs and CRISPR/Cas9

gRNAs were designed and constructed to target top hits derived from pooled screen. The synthesized oligos were listed in Table S4 (Supporting Information). Hct116 cells were transduced with lentivirus containing gRNAs as described above. Single‐cell clones were isolated by FACS (BD FACSAria III), including a gate to exclude doublets, to generate three 96‐well plates per guide. Approximately 20% of the wells yielded viable cells. After 14 d expansion, clones were subjected to PCR amplification and mutation analysis. Genomic loci containing the corresponding gRNA‐targeted DNA sequences were PCR amplified using the primer pairs in Table S6 (Supporting Information) and sequenced by Sanger. Sequencing traces were compared with expected single‐base substitutions. Mutant clones consistent with expected mutations in Table S3 (Supporting Information) were expanded for subsequent experiment. To obtain P53 KO Hct116 cells, two sgRNAs (*TP53*‐sgRNA1: cgtcgagccccctctgagtc; *TP53*‐sgRNA2: cccttcccagaaaacctacc) were constructed to target the coding region of *TP53*. The two sgRNAs were transiently transfected into Hct116 cells, and single cell clones were expanded and genotyped using a pair of primers (*TP53* forward: ctcagacactggcatggtgtt; *TP53* reverse: atacggccaggcattgaagtc).

### RNA Isolation and Bulk RNA‐Seq Analysis

Cells were treated with TRIzol reagent (Vazyme, R401‐01) and then RNA was extracted using EasyPure Kit (Transgene, R101‐1), according to the manufacturer's instructions. 500 ng total RNA was subjected to bulk RNA‐seq using Illumina Nova‐seq platform (Novogene, China) at a depth of ≈20 million reads per sample. RNA‐seq data analysis was conducted as previously described.^[^ ^47^ ^]^ The paired‐end RNA‐seq reads were mapped to the GRCh38/hg38 reference genome by using STAR (v2.5.3a). Gene expression was quantified to FPKM for each gene using RSEM (v1.3.0). Raw read counts were calculated by featureCounts (v1.5.2). Differential gene expression analysis was performed using DESeq2 (v1.22.1). Heatmaps were generated using the FPKM values using R package pheatmap (v1.0.10). Gene ontology (GO) and Kyoto Encyclopedia of Genes and Genomes (KEGG) pathway analysis was performed using R/Bioconductor package clusterProfiler (v3.10.0). Gene set enrichment analysis (GSEA) was performed using an online tool (http://www.webgestalt.org/).

### Apoptosis Analysis

Cells treated with or without chemical reagents were stained with annexin V‐fluorescein isothiocyanate (FITC)/PI kit for apoptotic analysis (A211‐02; Vazyme, China). At indicated time points, the cells were centrifuged at 1000 rpm for 5 min and the supernatant was removed. 100 µL Annexin‐V binding buffer was added to resuspend the cells and cells were stained at dark using 5 µL Annexin‐V‐FITC and 5 µL PI for 15 min at room temperature. Then, 150 µL Annexin‐V binding buffer was added into the reaction system and the samples were immediately analyzed using a flow cytometer (FACS Caliber, Becton‐Dickinson). Compensation was conducted for each experiment using untreated cells stained with Annexin V and PI. Cells without staining or staining with only Annexin‐V‐FITC or PI served as controls.

### Cell Proliferation Assay

CCK‐8 assay was performed to determine the cell viability via a Cell Counting Kit‐8 (CCK‐8) (Vazyme, China). After washing with PBS, Hct116 cells were trypsinized into single cells and suspended with PBS. Then 20 µL cell suspension was added into 20 µL trypan blue for cell counting. Cells were plated into 96‐well plates (10 000 cells per well), and CCK‐8 assay was performed at indicated time points. Briefly, cells were incubated with CCK‐8 solution for 3 h, and then the stop solution was added into the medium. The optical density values (OD) were determined at 450 nm utilizing a fully functional microporous plate detector (envision; PerkinElmer, Shanghai). Three independent replicates were performed and presented.

### Mass Spectrum Analysis of Total and Phosphorylated Proteins

DMSO‐ or 5‐FU‐treated Hct116 cells (5‐FU, 10 × 10^−6^ m for 24 h; 2 × 10^7^) were collected for mass spectrum analysis. Sample was sonicated three times on ice using a high‐intensity ultrasonic processor in lysis buffer (8 M urea, 1% Protease Inhibitor Cocktail). The remaining debris was removed by centrifugation at 12 000 g at 4 °C for 10 min. Finally, the supernatant was collected, and the protein concentration was determined with BCA kit (Thermofisher). Extracted total proteins were subjected to Coomassie brilliant blue staining after separation on 10% SDS‐PAGE gel. Then, total protein was subjected to trypsin digestion, bio‐material‐based (IMAC microspheres suspension) PTM enrichment for phosphorylation‐modified peptides, and LC‐MS/MS analysis. For total protein detection, the tryptic peptides were directly subjected to LC‐MS/MS analysis without modification enrichment. Briefly, the tryptic peptides were dissolved in 0.1% formic acid (solvent A), directly loaded onto a home‐made reversed‐phase analytical column. The gradient was comprised of an increase from 6% to 23% solvent B (0.1% formic acid in 98% acetonitrile) over 26 min, 23% to 35% in 8 min and climbing to 80% in 3 min then holding at 80% for the last 3 min, all at a constant flow rate of 400 nL min^−1^ on an EASY‐nLC 1000 UPLC system. The peptides were subjected to NSI source followed by tandem mass spectrometry (MS/MS) in Q ExactivePlus (Thermofisher) coupled online to the UPLC. Peptides were then selected for MS/MS using NCE setting as 28 and the fragments were detected in the Orbitrap at a resolution of 17500. A data‐dependent procedure that alternated between one MS scan followed by 20 MS/MS scans with 15.0 s dynamic exclusion. The resulting MS/MS data were processed using Maxquant search engine (v.1.5.2.8) and subjected to data search and bioinformatic analysis.

### Western Blot Analysis

Collected cell samples were lysed by RIPA lysis buffer (Beyotime, China) on ice for 30 min, and then lysis mixture was centrifugated at 12 000 rpm for 10 min. The supernatant was harvested, and the concentration was assessed using a BCA kit. Samples were diluted to a same concentration and added 5× SDS loading buffer according to the volume of the lysate, and samples were boiled at 100 °C for 5 min. Then, specimens were separated on 8–12% SDS‐PAGE and transferred onto PVDF membranes. The PVDF membranes were blocked with 5% milk/TBST at room temperature for 1 h. Primary antibodies against p‐RSK2 (T577) (1:500; sc‐374664, Santa Cruz), RSK2 (1:500; sc‐9986, Santa Cruz), GAPDH (1:20000; 60004‐1‐lg, Proteintech), P53 (1:20000;10442‐1‐AP, Proteintech), and p‐*γ*H2Ax (S139) (1:1000; #9718, Cell Signaling), were diluted with 1% BSA/TBST and incubated with PVDF membranes overnight at 4 °C. The PVDF membranes were then incubated with HRP‐labeled goat anti‐rabbit (1:5000; Thermofisher) or anti‐mouse secondary antibodies (1: 5000; Thermofisher) diluted by 5% milk/TBST at room temperature for 1 h. The membranes were then subjected to ECL imaging using ECL kit (Vazyme, China), and images were captured with Tannon 5200SF at an auto mode.

### Phosphorylation Network Analysis and Gene Expression Analysis in Pan‐Cancers

Top 20 screen hits were subjected to phosphorylation network analysis using an online tool (http://phosphorylationnetworks.org). Briefly, top screen hits were uploaded as input and analysis program was executed. The expression of *RPS6KA3* and *PAK4* was performed using Gene Expression Profiling Interactive Analysis (GEPIA) 2 (http://gepia2.cancer‐pku.cn/#index).

### Statistical Analysis

Statistical analysis was performed using GraphPad Prism 8.0, and Student's *t* test was performed for statistical analysis. *P < 0 .05, **P < 0.01, ***P < 0 .001, and ****P < 0.0001. Results were obtained from 2 or 3 independent experiments, each with three replicates. Data were presented as the mean ± SD values.

## Conflict of Interest

The authors declare no conflict of interest.

## Author Contributions

J.N.L., J.X.L., and S.H. contributed equally to this work. J.N.L., J.X.L., and Y.Q. designed and performed the experiments. S.H. and M.L. performed computational analysis and validating experiments. W.Y., Y.Z., and J.G. helped with the data analysis. P.Z., Y.Q., and X.H. designed, conceived, and supervised the work and co‐wrote the manuscript.

## Supporting information

Supporting Information

Supporting Information

Supporting Information

Supporting Information

Supporting Information

Supporting Information

Supporting Information

## Data Availability

The data that support the findings of this study are available from Sequence Read Archive. Restrictions apply to the availability of these data, which were used under license for this study. Data are available at https://dataview.ncbi.nlm.nih.gov/object/PRJNA783166?reviewer=ls2pmo2r2dee12tu9fd5p477re with the permission of Sequence Read Archive.
