# Supplementary material for: Functional Phosphoproteomics in Cancer Chemoresistance Using CRISPR‐Mediated Base Editors
Source: Adv Sci (Weinh). 2022 Aug 31;9(30):2200717. doi: 10.1002/advs.202200717 (PMC9596822; doi:10.1002/advs.202200717)
Supplement: Supplementary file 1 — Supporting Information [file ADVS-9-2200717-s002.pdf]

ADVANCED SCIENCE
Open Access

Supporting Information

for *Adv. Sci.*, DOI 10.1002/advs.202200717

Functional Phosphoproteomics in Cancer Chemoresistance Using CRISPR-Mediated Base Editors

Jianan Li, Jianxiang Lin, Shisheng Huang, Min Li, Wenxia Yu, Yuting Zhao, Junfan Guo, Pumin Zhang, Xingxu Huang and Yunbo Qiao**

Copyright WILEY-VCH Verlag GmbH & Co. KGaA, 69469 Weinheim, Germany, 2020.

Supporting Information

Functional phosphoproteomics in cancer chemoresistance using CRISPR-mediated base editors

Jianan Li, Jianxiang Lin, Shisheng Huang, Min Li, Wenxia Yu, Yuting Zhao, Junfan Guo, Pumin Zhang, Xingxu Huang, and Yunbo Qiao**

Supplementary Figures and Tables:

Contents

Figure S1 2
Figure S2 4
Figure S3 5
Figure S4 7
Figure S5 9
Figure S6 10
Figure S7 11
Figure S8 13
Figure S9 14
Figure S10 15
Figure S11 16
Figure S12 18
Figure S13 20

Figure S1

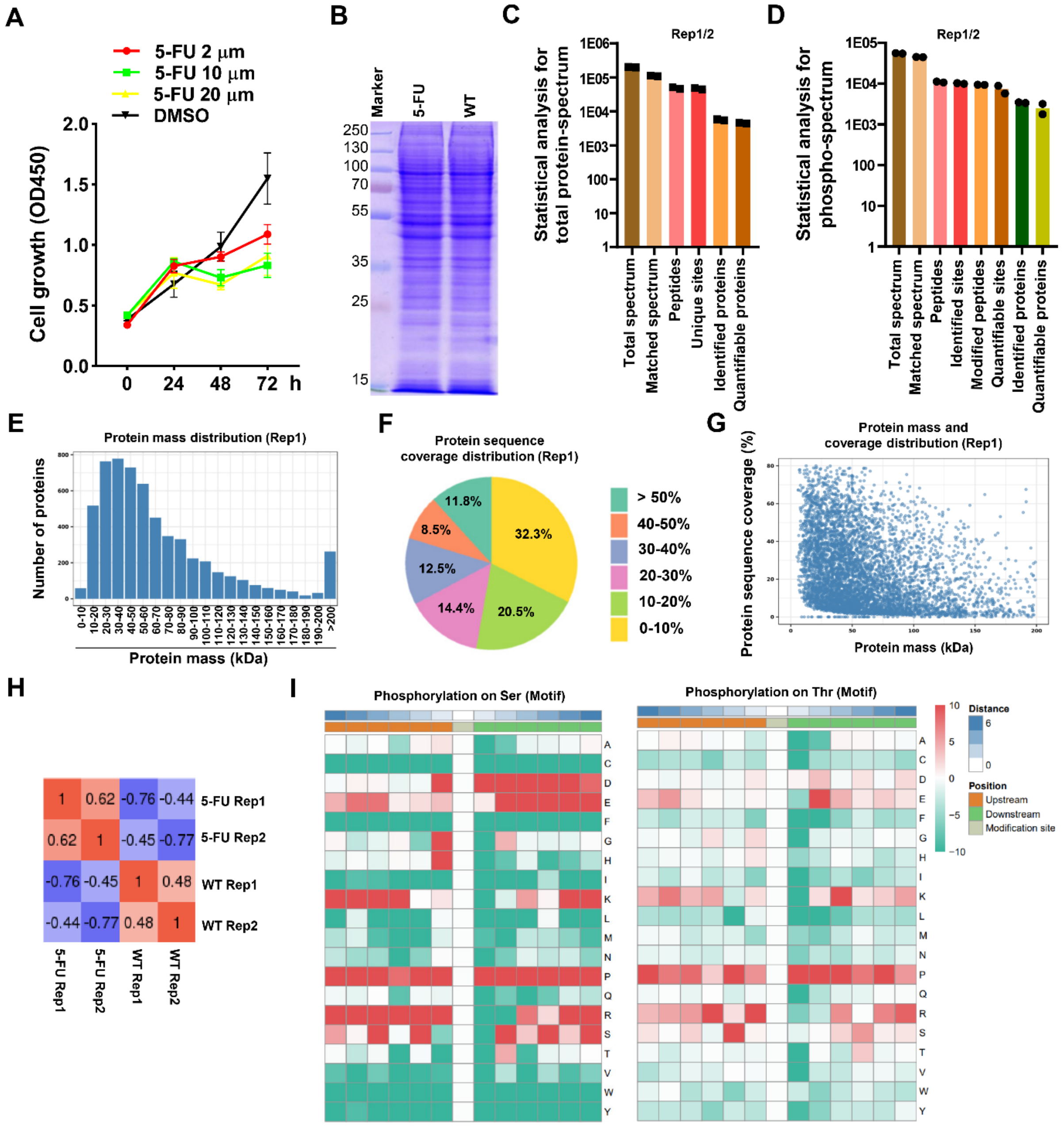

Figure S1. Proteomics analysis of proteins in control and 5-FU-treated Hct116 cells.

A) Cell proliferation analysis of DMSO- or 5-FU-treated Hct116 cells using CCK8 assays. The OD450 absorbance values were evaluated and normalized to calculate cell proliferation rates at 0, 24, 48, and 72 h.

B) Visualization of proteins separated by SDS-PAGE (10%) using Coomassie brilliant blue staining of DMSO- or 5-FU-treated Hct116 cells.

C) Statistical analysis of total proteins using mass spectrum analysis with two replicates (Rep1/2).

D) Statistical analysis of phosphorylated proteins using mass spectrum analysis with two replicates (Rep1/2). Phosphorylated proteins were enriched by using pan-phosphorylation antibodies.

E) The number of proteins with different ranges of masses was calculated (Rep1 as an example).

F) The protein sequence coverage distributions for detected proteins (Rep1 as an example). Over 50% of proteins were covered for less than 20% (0-10%, 32.3%; 10-20%, 20.5%).

G) The protein mass and sequence coverage distributions for detected proteins (Rep1 as an example).

H) Clustering analysis of detected proteins between DMSO- or 5-FU-treated Hct116 cells with two replicates for each group.

I) Protein sequence preference for the upstream (-6) or downstream (+6) of phosphorylated Ser and Thr.

Figure S2

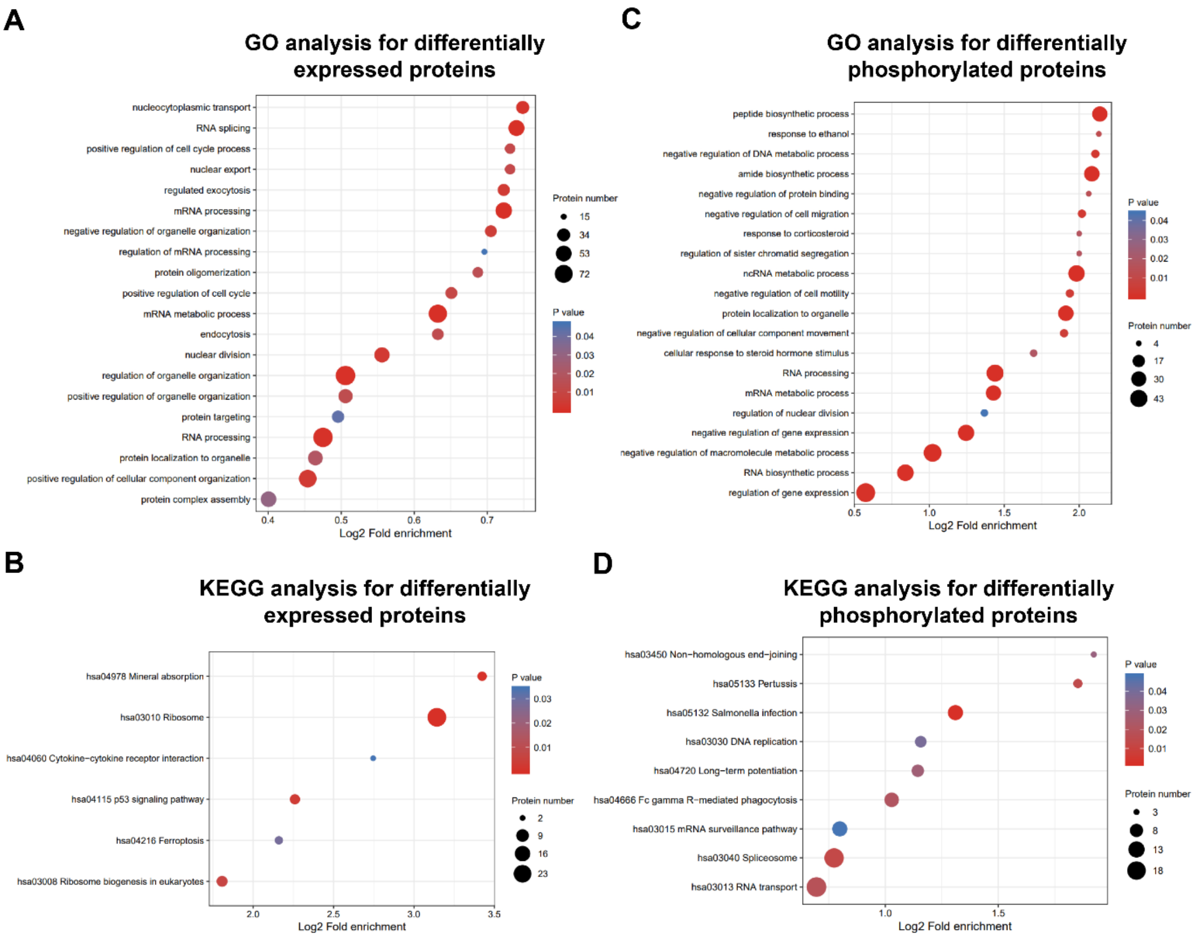

Figure S2. Analysis of differentially expressed proteins upon 5-FU treatment.

A-B) Gene ontology (GO) (A) and KEGG pathway (B) analysis of differentially expressed proteins upon 5-FU treatment (FC > 2).

C-D) Gene ontology (GO) (C) and KEGG pathway (D) analysis of differentially phosphorylated proteins upon 5-FU treatment (FC > 2).

Figure S3

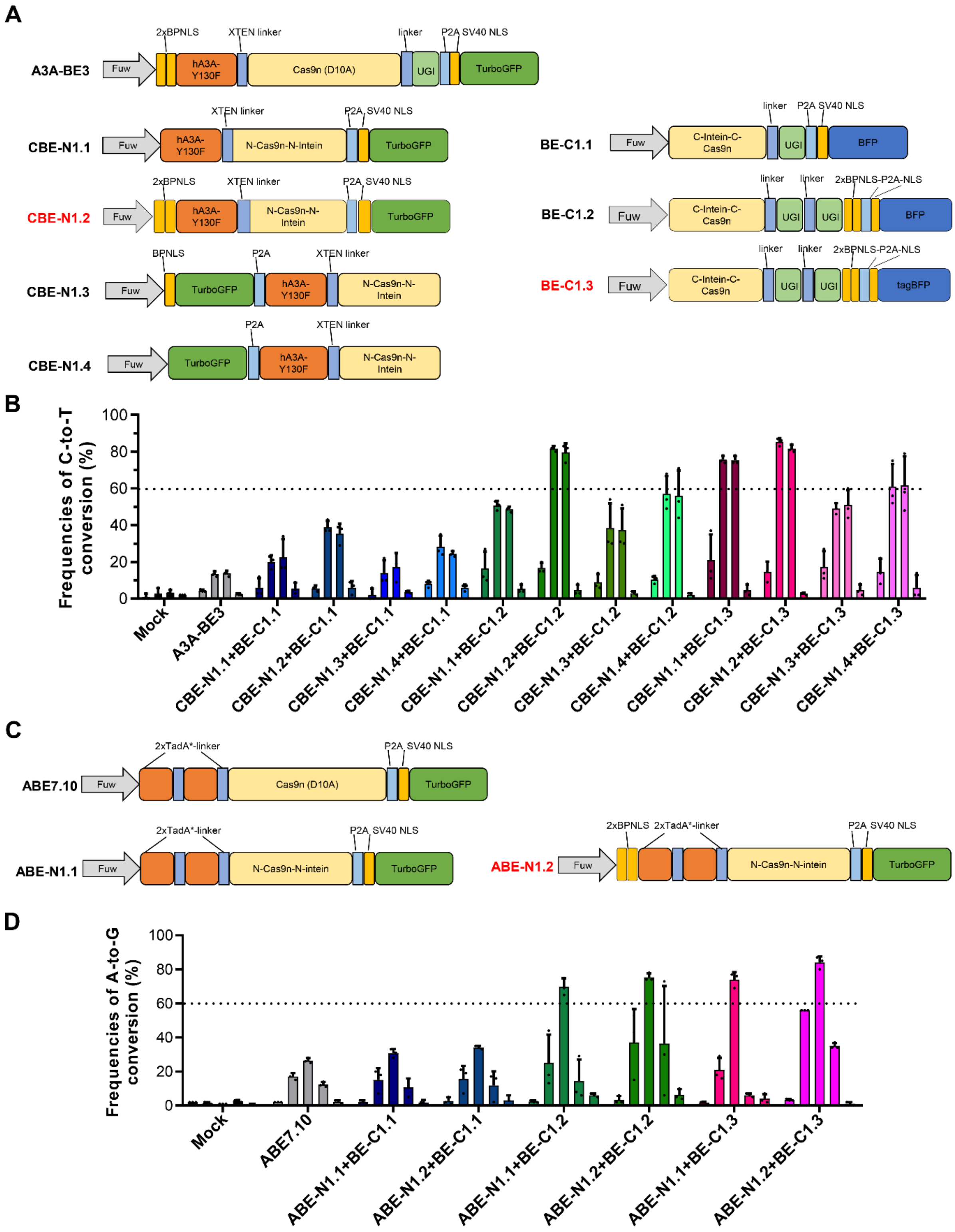

Figure S3. Construction of split-BE system for highly efficient base editing.

A) The schematic diagram showing the structures of full length or split A3A-BE3. Four versions (CBE-N1.1 to N1.4) of N-terminal part of A3A-BE3 were constructed, all of which

contain human APOBEC3A (hA3A)-Y130F, N-Cas9n (1-573 aa) and N-Intein, with TurboGFP and NLS located at indicated positions. Three versions (BE-C1.1 to C1.3) of C-terminal part of A3A-BE3 were constructed, containing C-Intein, C-Cas9n (574-1382 aa), one or two repeats of UGI, and BFP (or tagBFP).

B) Sanger sequencing analysis of C-to-T conversion efficiencies in HEK293T cells transfected with indicated combinations of split cytosine base editors.

C) The schematic diagram showing the structures of full length or N-terminal of split ABE.

D) Sanger sequencing analysis of A-to-G conversion efficiencies in HEK293T cells transfected with indicated combinations of split ABEs. It is worth noticing that the C-terminal part of split ABE was same to the C-terminal part of split A3A-BE3 in (A).

Figure S4

A

Modification Type	Number	Base editors			Nonconvertible	Convertible	Alterations of amino acid types								
		ABE	CBE	ABE&CBE			A-to-A	A-to-B	A-to-N	B-to-A	B-to-B	B-to-N	N-to-A	N-to-B	N-to-N
**Phosphoserine**	29659	**8075**	**6715**	1961	16830	12829	0	0	0	0	0	0	0	0	**12829**
N-linked glycosylation	14237	3626	0	0	10611	3626	0	0	0	3	0	5	1213	0	3423
**Phosphothreonine**	5313	**1233**	**1064**	738	3754	1559	0	0	0	0	0	0	0	0	**1559**
Sumoylation	5089	998	0	0	4091	998	0	0	0	323	297	794	0	0	0
Lysine acetylation	3929	817	0	0	3112	817	0	0	0	256	249	659	0	0	0
**Phosphotyrosine**	1911	**787**	**0**	0	1124	787	0	0	0	0	0	0	0	**477**	**431**
Methylation	1802	194	471	37	1174	628	0	0	0	11	197	453	1	25	20
non-lysine acetylation	1404	100	529	37	812	592	0	0	0	0	0	0	1	3	591
N6-succinyllysine	1191	239	0	0	952	239	0	0	0	65	85	192	0	0	0
Others	1107	196	124	20	807	300	0	12	30	14	31	85	21	79	99
O-linked glycosylation	971	226	184	90	651	320	0	0	0	2	8	7	0	3	307
Hydroxylation	954	92	119	0	743	211	0	0	4	17	28	50	10	0	137
Ubiquitylation	546	120	1	0	425	121	0	0	0	32	39	97	0	0	1
Palmitoylation	428	93	97	64	302	126	0	0	0	0	0	0	0	93	97
Geranyl-geranylation	151	43	38	24	94	57	0	0	0	0	0	0	0	43	38
Amidation and Deamidation	150	31	34	7	92	58	0	0	4	0	0	0	8	5	48
Nitration	131	37	11	8	91	40	0	0	0	0	0	0	0	26	28
ADP-ribosylation	86	13	12	2	63	23	0	4	2	0	1	7	0	1	9
Pyrrolidone Carboxylic Acid	78	19	0	0	59	19	0	0	0	0	0	0	0	19	0
C-linked Glycosylation	63	12	0	0	51	12	0	0	0	0	0	0	0	12	0
Phospho-others	60	12	5	1	44	16	0	0	0	5	1	14	1	0	2
Farnesylation	58	14	7	5	42	16	0	0	0	0	0	0	0	14	7
Sulfation	23	9	7	7	14	9	0	0	0	0	0	1	0	6	8

A, acidic ; B, basic; N, neutral

B

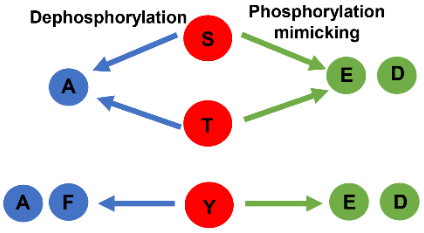

C

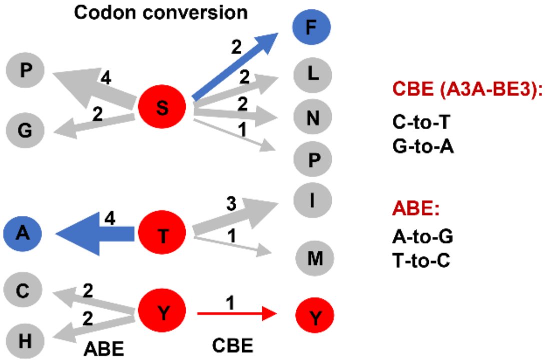

D

Phosphorylation site	Converted to	ABE	CBE	ABE&CBE
S	P,L	410	410	410
S	G,N	768	768	768
S	P,F	783	783	783
S	L	0	994	0
S	F	0	1690	0
S	G	1976	0	0
S	N	0	2070	0
S	P	4138	0	0
T	M	0	41	0
T	A,M	110	110	110
T	I	0	285	0
T	A	495	0	0
T	A,I	628	628	628
Y	C,H	121	0	0
Y	C	310	0	0
Y	H	356	0	0
Total		10095	7779	

Figure S4. Information for split-A3A-BE3 and -ABE library design.

A) Summary for post-translational modification types and corresponding number of modification sites. The number of modification sites that can be converted into another amino

acids by using ABE or CBE or both was also presented. The alterations of the characteristics of amino acids (A, acidic; B, basic; N, neutral) before or after conversions were calculated.

B) The expected conversions of phosphorylated amino acids (S, T, and Y) into other kinds of amino acids to mimic dephosphorylation or phosphorylation status.

C) The potential conversion results induced by CBE or ABE with 1-4 kinds of codon conversions.

D) The detailed information for final constructed CBE or ABE libraries. The types of converted amino acids were calculated.

Figure S5

A

CBE (A3A-BE3) gRNA library design:

6,828 target site---6,828 gRNA
951 target site---2,057 gRNA
400 non-target control gRNA
→ **9,285 gRNA**

ABE gRNA library design:

10,041 target site---10,041 gRNA
54 target site---108 gRNA
705 non-target control gRNA
→ **10,854 gRNA**

B

QC by NGS	Reads	gRNA number	Detected gRNA	Missed gRNA	Coverage	Homogeneity
**CBE gRNA library**	2,096,358	9,285	9,284	1	99.99%	4.56
**ABE gRNA library**	3,409,123	10,854	10,854	0	100%	3.56

C

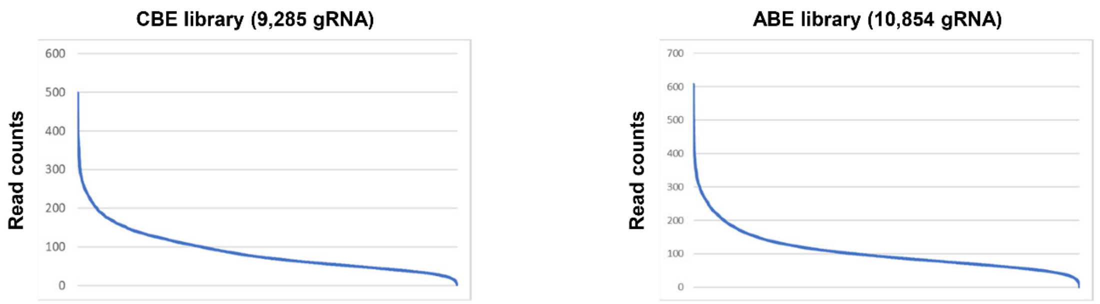

Figure S5. Quality control of constructed libraries.

A) Summary for constructed CBE and ABE libraries, including target sites with a single gRNA, target sites with 2 or 3 gRNAs, and non-target control gRNAs.

B) Next generation sequencing (NGS) analysis of CBE and ABE libraries. The quality of the two libraries was evaluated.

C) The read count distributions of each gRNA for CBE and ABE libraries.

Figure S6

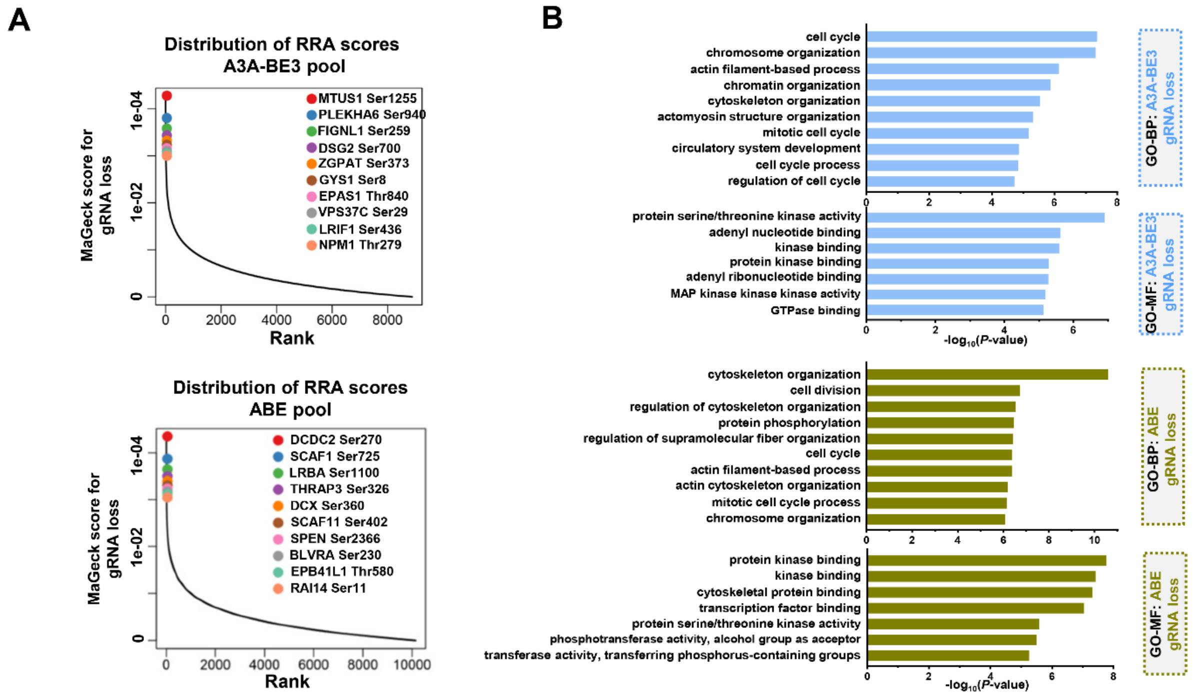

Figure S6. Analysis of gRNA loss upon 5-FU treatment.

A) Top gRNAs that were lost during 5-FU treatment relative to gRNAs expressed in control Hct16 cells using MAGeCK algorithm (ranked by MAGeCK RPA scores).

B) GO analysis of genes targeted by gRNAs that were significantly lost upon 5-FU treatment. Two types of GO terms, including biological process (BP) and molecular function (MF), were presented.

Figure S7

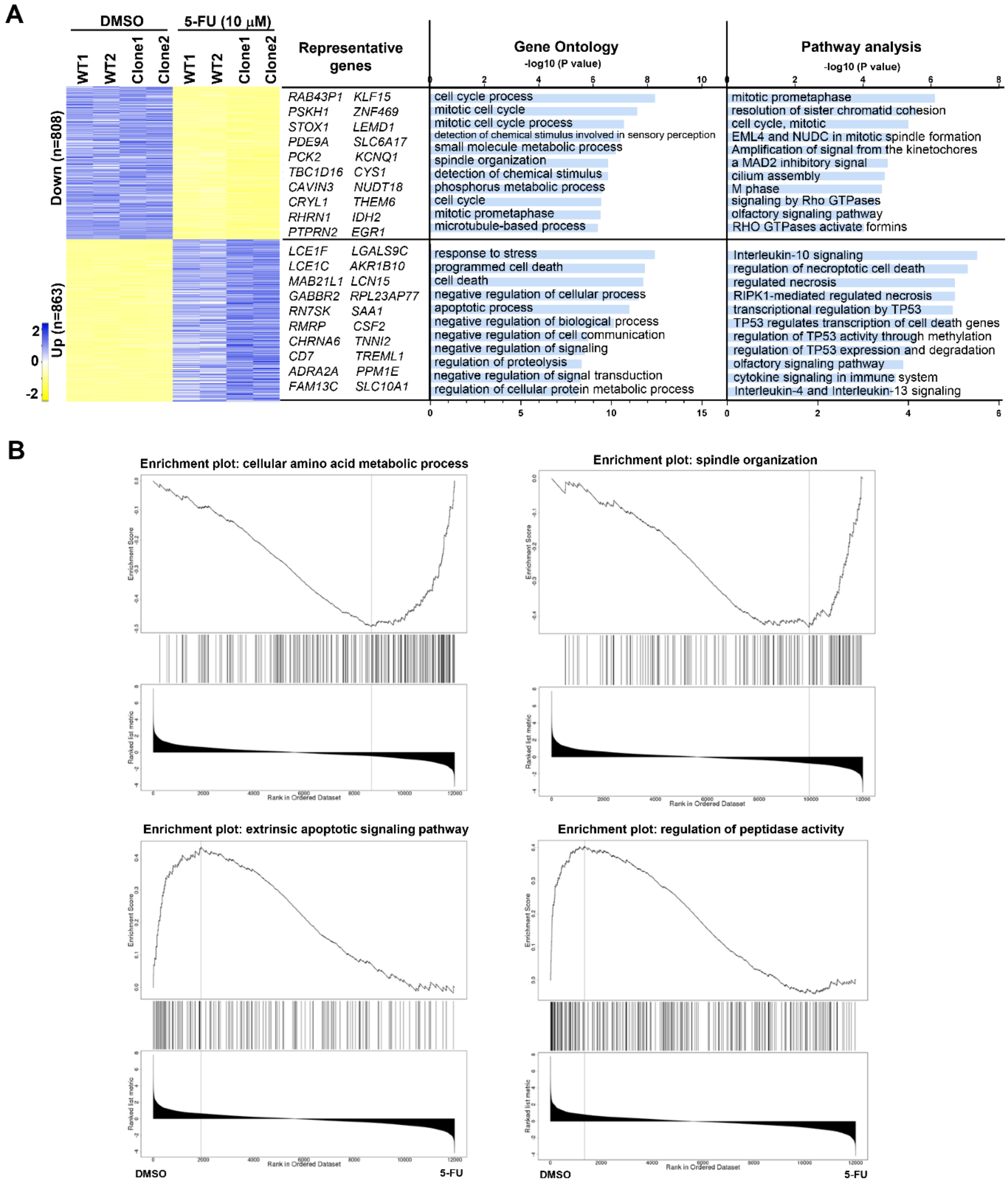

Figure S7. Analysis of 5-FU-triggered differentially expressed genes.

A) RNA-seq analysis of DMSO- and 5-FU-treated Hct116 cells. Two replicates treated with DMSO or 5-FU (WT1 and WT2) and two single clones separated from Hct16 cell lines were subjected to this experiment. Differentially expressed genes (Down and Up) were presented,

and representative genes were displayed. Gene ontology and KEGG pathway analyses were also performed, and top enriched terms with P values were shown.

B) Gene set enrichment analysis (GSEA) was performed for upregulated or downregulated genes elicited by 5-FU treatment.

Figure S8

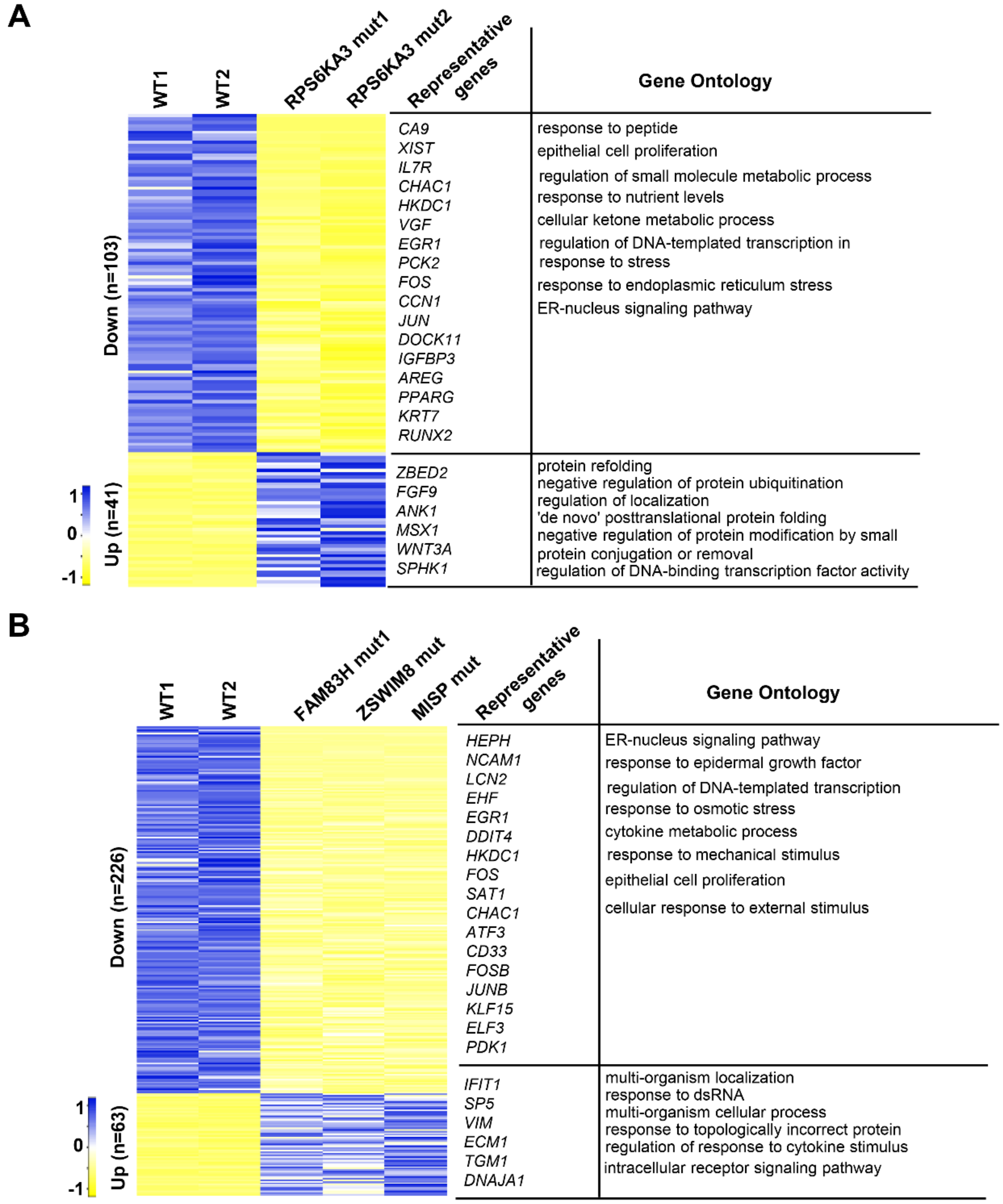

Figure S8. Analysis of 5-FU-triggered differentially expressed genes.
A-B) RNA-seq analysis of control Hct116 (WT1 and WT2) cells and RPS6KA6 mutant cells (two single clones, mut1 and mut2) (A) or FAM83H, ZSWIM8, and MISP mutant cells (B). Differentially expressed genes were shown as a heatmap, and representative genes were displayed. Gene ontology analysis was performed, and top enriched terms were shown.

Figure S9

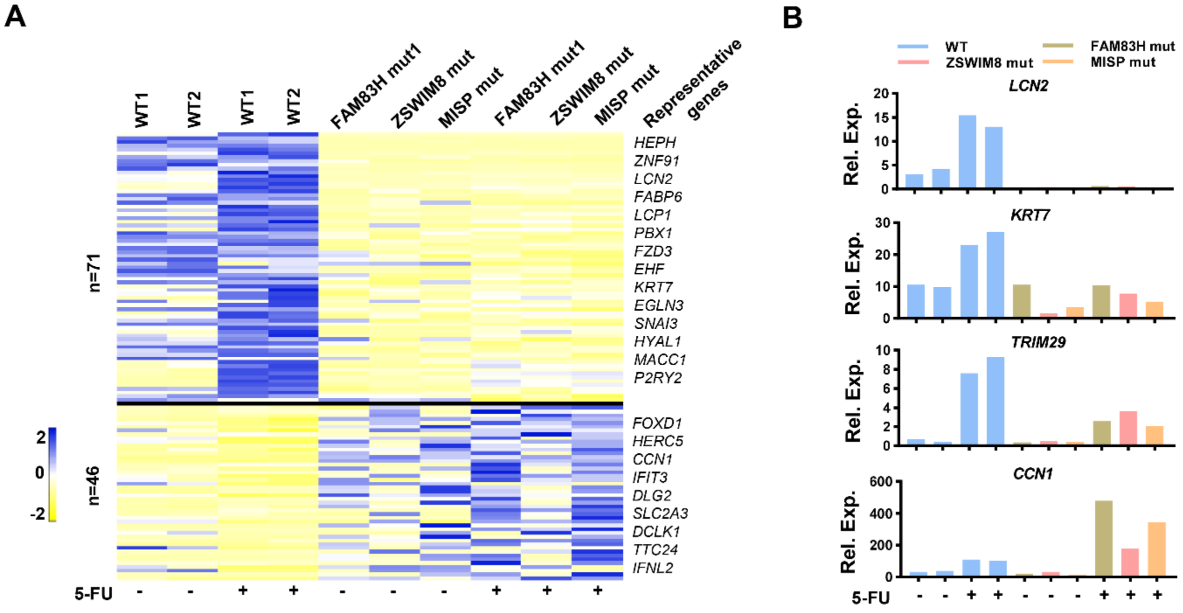

Figure S9. Analysis of FAM83H, ZSWIM8, and MISP mutant cells in responding to 5-FU treatment.

A) RNA-seq analysis of FAM83H, ZSWIM8, and MISP mutant cells relative to control Hct116 cells (WT1 and WT2) with or without 5-FU treatment. The genes were differentially expressed in control and mutant cells treated with 5-FU. Representative genes were presented.

B) Relative expression (Rel. Exp.) of *LCN2*, *KRT7*, *TRIM29*, and *CCN1* in control Hct116 cells (WT1 and WT2) and FAM83H, ZSWIM8, and MISP mutant cells with or without 5-FU treatment.

Figure S10

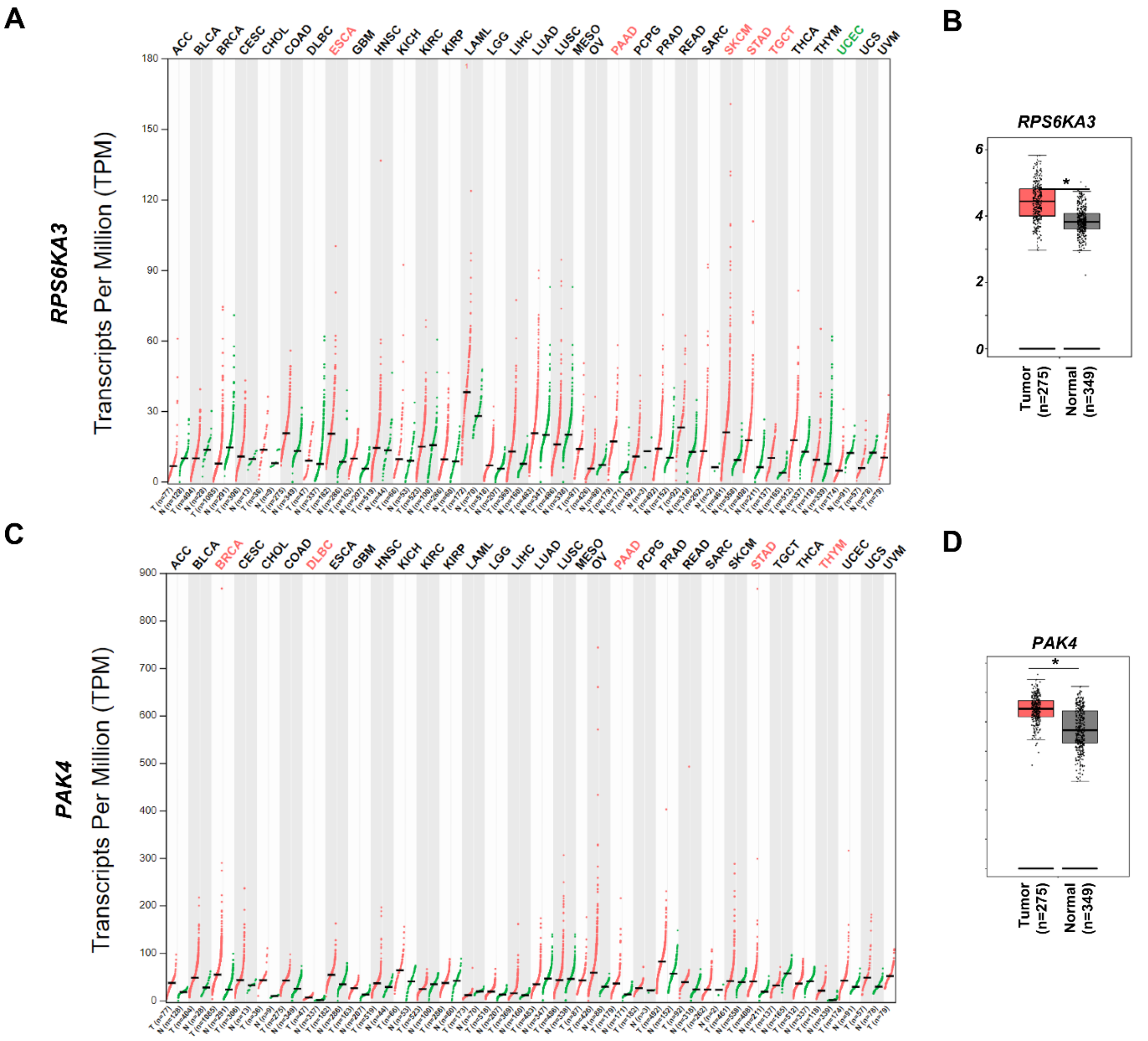

Figure S10. Expression of *RPS6KA3* and *PAK4* in pan-cancers.

A) Analysis of *PRS6KA3* expression in tumor (T) and normal (N) tissues in pan-cancers using GEPIA2 (http://gepia2.cancer-pku.cn/).

B) Analysis of *PRS6KA3* expression in tumor and normal tissues in colorectal cancer.

C) Analysis of *PAK4* expression in tumor (T) and normal (N) tissues in pan-cancers using GEPIA2.

D) Analysis of *PAK4* expression in tumor and normal tissues in colorectal cancer.

Figure S11

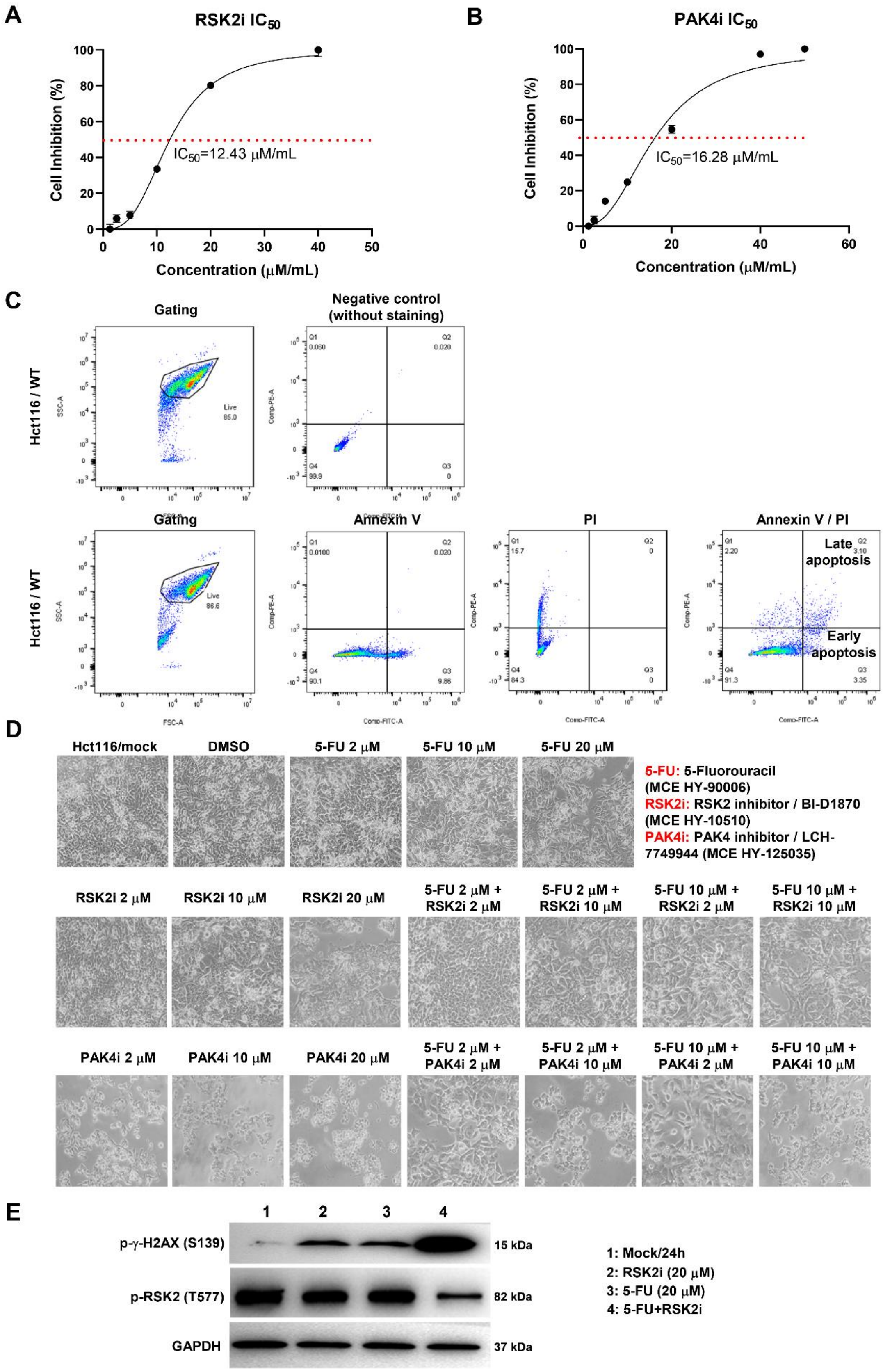
A
RSK2i IC50
Cell Inhibition (%)
IC50=12.43 μM/mL
Concentration (μM/mL)
B
PAK4i IC50
Cell Inhibition (%)
IC50=16.28 μM/mL
Concentration (μM/mL)
C
Gating
Negative control (without staining)
Hct116 / WT
Annexin V
PI
Annexin V / PI
Late apoptosis
Early apoptosis
D
Hct116/mock
DMSO
5-FU 2 μM
5-FU 10 μM
5-FU 20 μM
5-FU: 5-Fluorouracil (MCE HY-90006)
RSK2i: RSK2 inhibitor / BI-D1870 (MCE HY-10510)
PAK4i: PAK4 inhibitor / LCH-7749944 (MCE HY-125035)
RSK2i 2 μM
RSK2i 10 μM
RSK2i 20 μM
5-FU 2 μM + RSK2i 2 μM
5-FU 2 μM + RSK2i 10 μM
5-FU 10 μM + RSK2i 2 μM
5-FU 10 μM + RSK2i 10 μM
PAK4i 2 μM
PAK4i 10 μM
PAK4i 20 μM
5-FU 2 μM + PAK4i 2 μM
5-FU 2 μM + PAK4i 10 μM
5-FU 10 μM + PAK4i 2 μM
5-FU 10 μM + PAK4i 10 μM
E
p-γ-H2AX (S139)
15 kDa
p-RSK2 (T577)
82 kDa
GAPDH
37 kDa
1: Mock/24h
2: RSK2i (20 μM)
3: 5-FU (20 μM)
4: 5-FU+RSK2i

Figure S11. Synergistic effect of 5-FU and RSK2 or PAK4 inhibitors.

A-B) IC50 determination for RSK2 inhibitor (RSK2i; BI-D1870) and PAK4 inhibitor (PAK4i; LCH-7749944) in Hct116 cells for cell growth inhibition.

C) Gating strategy for apoptosis assays using Annexin V-FITC/PI Apoptosis Detection Kit (Vazyme). A negative control without Annexin V and PI staining was presented. Fluorescence compensation was executed before sample analysis.

D) Cell morphologies for Hct116 cells treated with increasing concentrations of 5-FU, RSK2 inhibitor, or PAK4 inhibitors at 48 h. Cells upon combinational treatment with 5-FU and RSK2i or PAK4i were also presented.

E) Western blot analysis of γ-H2Ax phosphorylation (S139), p-RSK2 (T577), and GAPDH in control and 5-FU and/or RSK2i- or PAK4i- treated cells at 24 h.

Figure S12

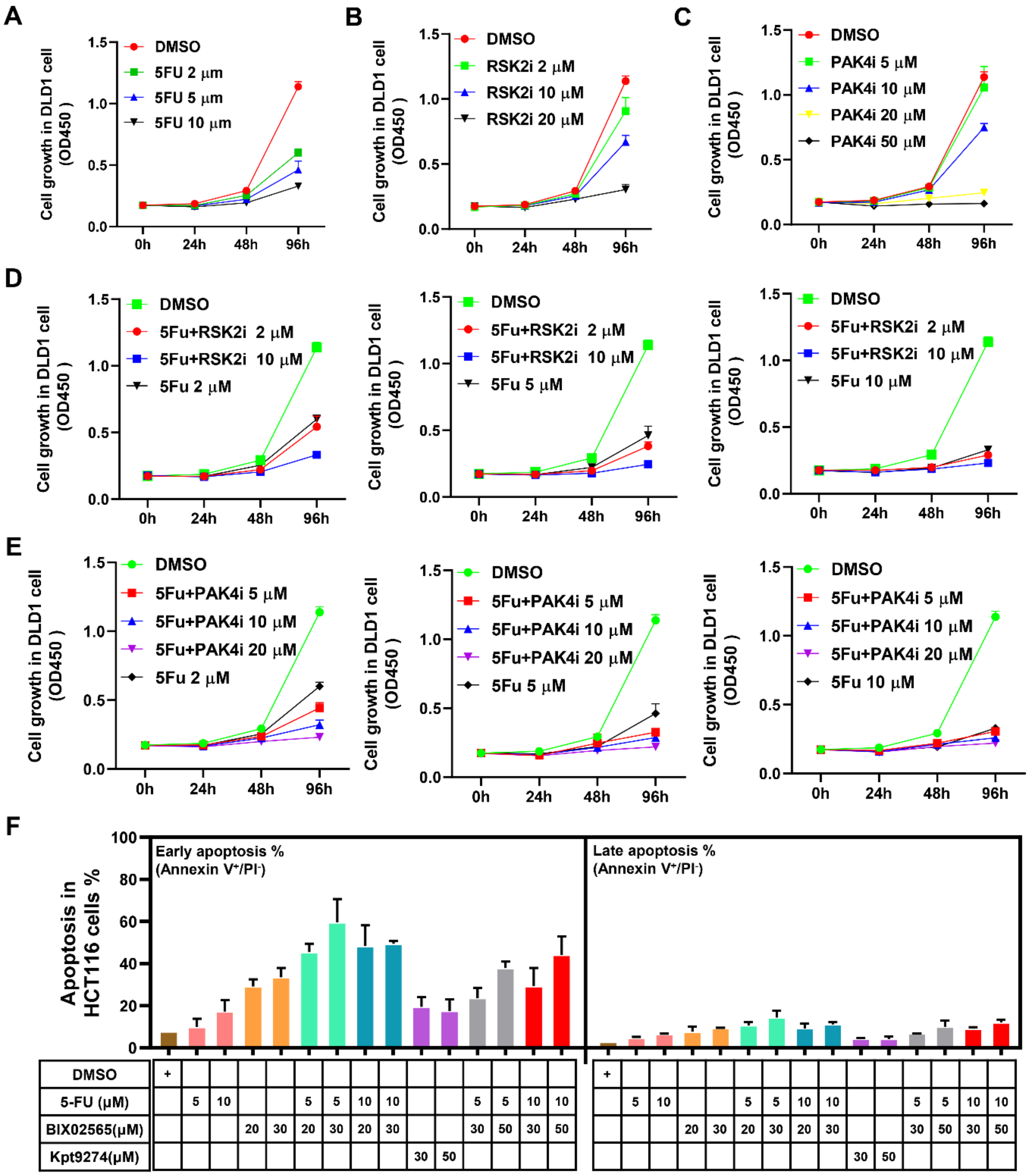

Figure S12. Synergistic effect of 5-FU and clinical RSK2 or PAK4 inhibitors.

A) The growth rate of DLD1 cells was determined upon DMSO or 5-FU (2, 5, 10 µM) at 0, 24, 48, and 96 h.

B) The growth rate of DLD1 cells was determined upon DMSO or RSK2 inhibitors (2, 10, 20 µM) at 0, 24, 48, and 96 h.

C) The growth rate of DLD1 cells was determined upon DMSO or PAK4 inhibitors (5, 10, 20, 50 μM) at 0, 24, 48, and 96 h.

D) The growth rate of DLD1 cells was determined upon combinational treatment with 5-FU (2, 5, or 10 μM) and RSK2 inhibitors (2 or 10 μM).

E) The growth rate of DLD1 cells was determined upon combinational treatment with 5-FU (2, 5, or 10 μM) and RSK2 inhibitors (5, 10, or 20 μM).

(F) Apoptotic analysis of Hct116 cells co-treated with 5-FU (2 or 10 μM) and BIX02565 (20 or 30 μM; a clinical RSK2 inhibitor) or Kpt9274 (30 or 50 μM; an orally bioavailable PAK4 inhibitor). Proportions of early (Annexin V^+/PI^-) or late (Annexin V^+/PI^+) apoptotic cells were presented.

Figure S13

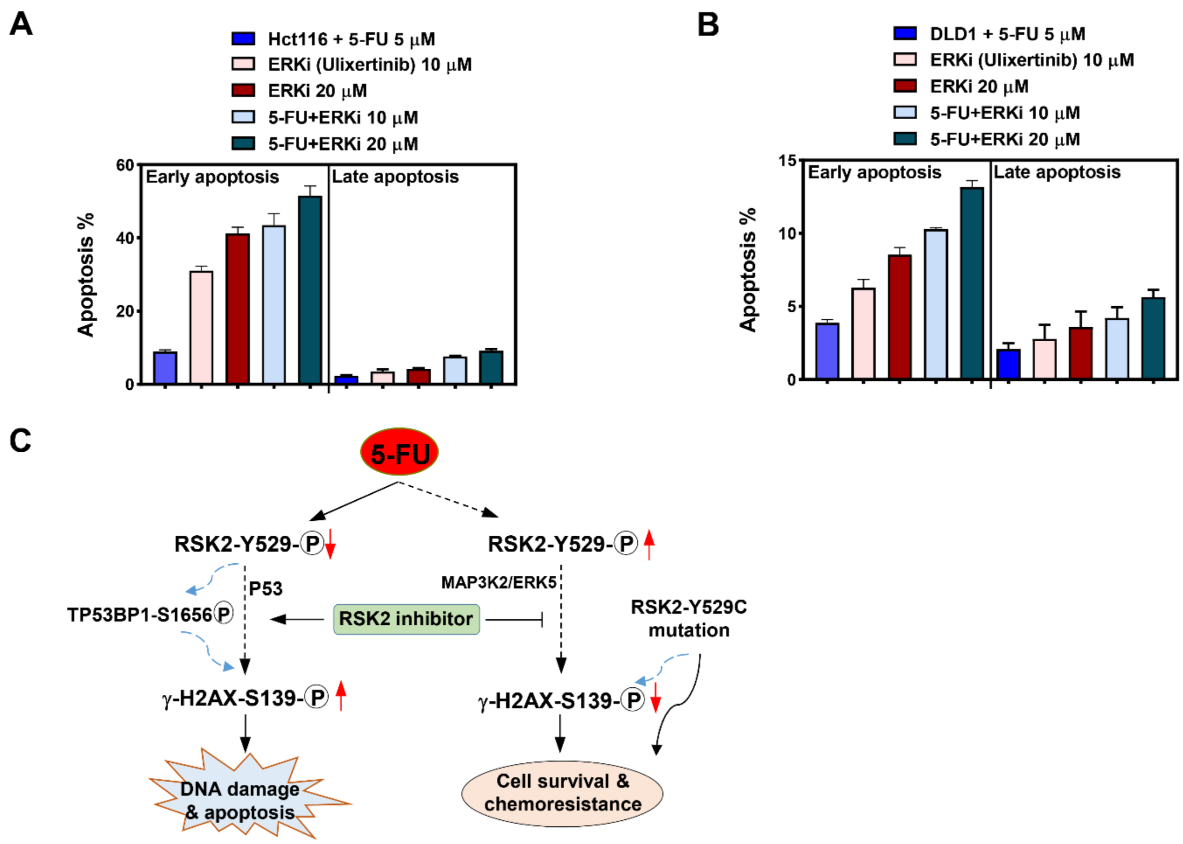

Figure S13. The role of ERK pathway in 5-FU-induced chemoresistance.

A-B) Apoptotic analysis of Hct116 cells (A) or DLD1 cells (B) treated 5-FU (5 μM) or ERK inhibitor (Ulixertinib; 10 or 20 μM) or their combinations. Proportions of early (Annexin V^+/PI^-) or late (Annexin V^+/PI^+) apoptotic cells were presented.

C) Working model for RSK2 functions in 5-FU-induced DNA damage and apoptosis as well as chemoresistance formation.
